# Pesticides impacts on human health and the environment with their mechanisms of action and possible countermeasures

**DOI:** 10.1016/j.heliyon.2024.e29128

**Published:** 2024-04-04

**Authors:** Md Faruque Ahmad, Fakhruddin Ali Ahmad, Abdulrahman A. Alsayegh, Md. Zeyaullah, Abdullah M. AlShahrani, Khursheed Muzammil, Abdullah Ali Saati, Shadma Wahab, Ehab Y. Elbendary, Nahla Kambal, Mohamed H. Abdelrahman, Sohail Hussain

**Affiliations:** a Department of Clinical Nutrition, College of Applied Medical Sciences, Jazan University, Jazan, Saudi Arabia; b Department of Basic and Applied Science, School of Engineering and Science, G.D Goenka University, Gururgram, Haryana, 122103, India; c Department of Basic Medical Science, College of Applied Medical Sciences, Khamis Mushayt Campus, King Khalid University (KKU), Abha, Saudi Arabia; d Department of Public Health, College of Applied Medical Sciences, Khamis Mushayt Campus, King Khalid University (KKU), Abha, Saudi Arabia; e Department of Community Medicine & Pilgrims Healthcare, Faculty of Medicine, Umm Al-Qura University, Saudi Arabia; f Department of Pharmacognosy, College of Pharmacy, King Khalid University, Abha, 62529, Saudi Arabia; g College of Applied Medical Sciences, Medical Laboratory Sciences, Jazan University, Jazan, 45142, Saudi Arabia; h Department of Pharmacology and Toxicology, College of Pharmacy, Jazan University, Jazan, 45142, Saudi Arabia

**Keywords:** Pesticides, Human health, Environment, Safety measures, Toxicology, Legislation

## Abstract

Pesticides are chemical constituents used to prevent or control pests, including insects, rodents, fungi, weeds, and other unwanted organisms. Despite their advantages in crop production and disease management, the use of pesticides poses significant hazards to the environment and public health. Pesticide elements have now perpetually entered our atmosphere and subsequently contaminated water, food, and soil, leading to health threats ranging from acute to chronic toxicities. Pesticides can cause acute toxicity if a high dose is inhaled, ingested, or comes into contact with the skin or eyes, while prolonged or recurrent exposure to pesticides leads to chronic toxicity. Pesticides produce different types of toxicity, for instance, neurotoxicity, mutagenicity, carcinogenicity, teratogenicity, and endocrine disruption. The toxicity of a pesticide formulation may depend on the specific active ingredient and the presence of synergistic or inert compounds that can enhance or modify its toxicity. Safety concerns are the need of the hour to control contemporary pesticide-induced health hazards. The effectiveness and implementation of the current legislature in providing ample protection for human health and the environment are key concerns. This review explored a comprehensive summary of pesticides regarding their updated impacts on human health and advanced safety concerns with legislation. Implementing regulations, proper training, and education can help mitigate the negative impacts of pesticide use and promote safer and more sustainable agricultural practices.

## 1 Introduction

Pesticides are natural or synthetic compounds that are applied to prevent, control, and eliminate insects, weeds, and pests that affect the growth of plants. These compounds are classified according to their mode of action, chemical structure, hazards, and application [1,2]. Since the mid-1940s, global pesticide demand has risen sharply and steadily, owing primarily to commercial farming [[3], [4], [5], [6]]. Excessive and uncontrolled pesticide use resulted in food contamination as well as environmental, agricultural, and aquatic pollution [7]. Fruits, vegetables, processed foods, water, air, and soil can all contain pesticide residues. Acute and chronic health effects from agricultural pesticides and dietary exposure are serious public health concerns, especially in developing countries. For human health, chemical pesticides can be carcinogenic, cytotoxic, and mutagenic [8]. Because pesticides mode of action is not specific to a single species, they frequently eradicate or harm organisms other than pests, including humans. According to a WHO and United Nations Environment Programme (UNEP) report, worldwide, three million people are poisoned and 200,000 die due to exposure to pesticides, mostly in developing countries [9]. Pesticides lead to the production of reactive oxygen species, which decrease the levels of antioxidants and their ability to protect cells from oxidative damage. Due to the imbalance, proteins, lipids, and nucleic acids affect cellular signaling pathways as well. Long-term health effects are caused by reactive oxygen species and oxidative stress [10]. Pesticides are frequently applied without precision, which leads to a number of adverse effects on human health, from acute intoxication to chronic diseases that include various types of cancer (brain cancer, breast cancer, prostate cancer, bladder cancer, and colon cancer) [11,12], Alzheimer's disease (AD) [13], Parkinson's disease [14], neurotoxicity [15,16], infertility [17,18], leukemia [19] and diabetes [20]. However, Pesticides have many advantages for agricultural productivity. They help to rise crop yields by decreasing losses caused by pests, diseases, and weeds [21,22]. They also contribute to food security by protecting the quantity and quality of harvested crops. In the field of public health, pesticides are employed to control disease-carrying insects like ticks, mosquitoes, and fleas, helping to avert the spread of diseases such as dengue fever, malaria, and Lyme disease [22,23]. Although the role of agrochemicals in increasing agricultural production is well established, the pesticide trade in the advanced world has seen substantial growth in the production and development of environmentally friendly pesticides with different formulations such as powder, solution, and emulsifiable concentrates [24,25]. Nevertheless, pesticides use must be balanced with environmental and health considerations, and responsible pesticide practices, such as integrated pest management, should be promoted to ensure sustainable and effective pest control strategies.

## 2 Materials and methods

For the current comprehensive review, we compiled evidence through diverse databases that included Science Direct, Scopus, the Saudi Digital Library, PubMed, and Google Scholar. The following keywords were used: pesticides, pesticide classification, pesticide effects on humans, pesticides and health hazards, pesticide impact on the environment, pesticide regulation, pesticide legislation, and pesticide safety. The following phrases were included: “pesticide effects on the central nervous system”, “pesticide effects on different types of cancer”, “effect of pesticides on infertility”, “effect of pesticides on respiratory disorders”, “effect of pesticides on diabetes”, “effect of pesticides on Alzheimer's disease”, “effect of pesticides on Parkinson's disease”, “effect of pesticides on asthma” and “pesticide exposure and safety measures”. Articles published in English were selected to study the consequences of pesticides on humans and the environment and possible safety measures.

## 3 Results and discussion

### 3.1 Classifications

Pesticides have been categorized into different groups based on their diverse applications and detrimental effects, which can be seen in the following sequences.

#### 3.1.1 Pesticide on the basis of chemical composition

Organochlorines, organophosphates, carbamates, and synthetic pyrethroids are the four core pesticides classified on the basis of chemical composition [2]. Organic insecticides with at least five chlorines are known as organochlorines or chlorinated hydrocarbons. Organochlorine pesticides are characterized by the presence of chlorine atoms in their chemical structure [26]. Pesticides containing organochlorines, for instance, DDT and chlordane, can produce convulsions and paralysis in the target pest, ending in death. Owing to their capacity to kill an extensive range of insects, this class of chemicals is utilized as an insecticide. Organophosphate pesticides have phosphorus and are derived from phosphoric acid. These are usually used as acaricides and insecticides. Organophosphates work by inhibiting the activity of cholinesterase, an enzyme essential for nerve function in insects. Examples include malathion, chlorpyrifos, and diazinon [27,28]. They can be found in soil, the atmosphere, and groundwater [[29], [30], [31]]. The organophosphate category includes parathion and malathion, which are usually connected to contact poison, and fumigant infection [32]. Although these insecticides have a moderate level of pest resistance, they are biodegradable, reducing pollution [32]. Carbamate pesticides are like to organophosphates in their mechanism but have a different chemical structure. Carbamates usually have a shorter environmental persistence compared to organophosphates. Examples include carbaryl, methomyl and propoxur [33,34]. Both insecticides kill target bugs by interrupting nerve signal transmission [35]. Environmental contamination can be reduced by allowing carbamates to spontaneously degrade. Synthetic pesticides, or pyrethroids, are a mixture of organic pesticides made by duplicating natural pyrethrins. Pyrethroids are non-persistent and simply break after exposure to light, making them the safest category of insecticides for food products. Neonicotinoids are a class of systemic insecticides that act on the nicotinic acetylcholine receptors in insects, causing paralysis and death. They are widely used in agriculture and are known for their effectiveness against sucking insects. Examples include imidacloprid, clothianidin, and thiamethoxam [36,37]. Similarly, glyphosate is a widely used herbicide that inhibits excitatory postsynaptic potential (EPSP). It obstruct the shikimic acid pathway, which plays a role in the production of aromatic amino acids in fungi, plants, and certain microbes [38,39]. Triazines are also a type of herbicide that impedes photosynthesis by targeting photosystem II. Several fungicides such as triazoles and strobilurins are ingested by the plant and transferred to various areas of the plant, such as leaves, stems, or interfering with critical metabolic functions [40,41].

#### 3.1.2 Pesticides on the basis of modes of entry

Pesticides can be classified into two main categories based on their modes of entry: systemic pesticides and non-systemic pesticides.

##### 3.1.2.1 Systemic pesticides

Systemic pesticides are a category of pesticide that is absorbed by plants and transported throughout their tissues, including stems, leaves, roots and flowers. Unlike contact pesticides that persist on the outward of the plant, systemic pesticides are used by the plant's vascular system and distributed internally [42,43]. Systemic insecticides are absorbed by plants and deliver a safeguard against insects that feed on the plant's tissues. Insects ingest the systemic pesticide when they consume sections of the treated plant and are injured or killed [44]. Systemic insecticides are mainly effective contrary to pests that have piercing-sucking mouthparts, as they can be controlled even if they are not directly exposed to the pesticide on the surface of plant [42]. Examples of systemic insecticides include imidacloprid, clothianidin, etc. Systemic pesticides have a high penetration capability in plant tissues, allowing them to flow either a unidirectional or multidirectional manner to kill target organisms [45]. The systemic nature of these pesticides provides various benefits. Even if the pesticide is only sprayed to a single area or place, it can protect the entire plant, including new growth. Because the pesticide is disseminated internally throughout the plant, systemic insecticides can also be effective against concealed or difficult-to-reach pests [46]. However, systemic pesticides can also be absorbed by non-target plants, which might have unexpected repercussions for beneficial insects or species that feed on treated plants.

##### 3.1.2.2 Non-systemic pesticides

Non-systemic pesticides are pesticides that do not migrate or translocate within the plant once they are applied. These pesticides remain on the plant's surface and are neither absorbed or dispersed within its tissues. Contact between target organisms and pesticides determines non-systemic insecticides [47]. Contact pesticides are administered directly to plant surfaces or to pests. They work by coming into contact with the pests and either killing or repelling them. Contact insecticides have efficacy against pests present at the time of application but may not give long-term protection [48]. Non-systemic pesticides are chiefly suitable when instant control or knockdown of pests is essential, and there is no need for longstanding residual protection [31,49]. Their effectiveness, however, may be limited to the sections of the plant that are directly treated, and they may need to be reapplied to new growth or when pests re-infest the treated region. When using non-systemic pesticides, it is critical to carefully follow the directions and safety precautions stated on the pesticide label [50].

##### 3.1.2.3 Pesticides on the basis of function

**Fig. 1:**
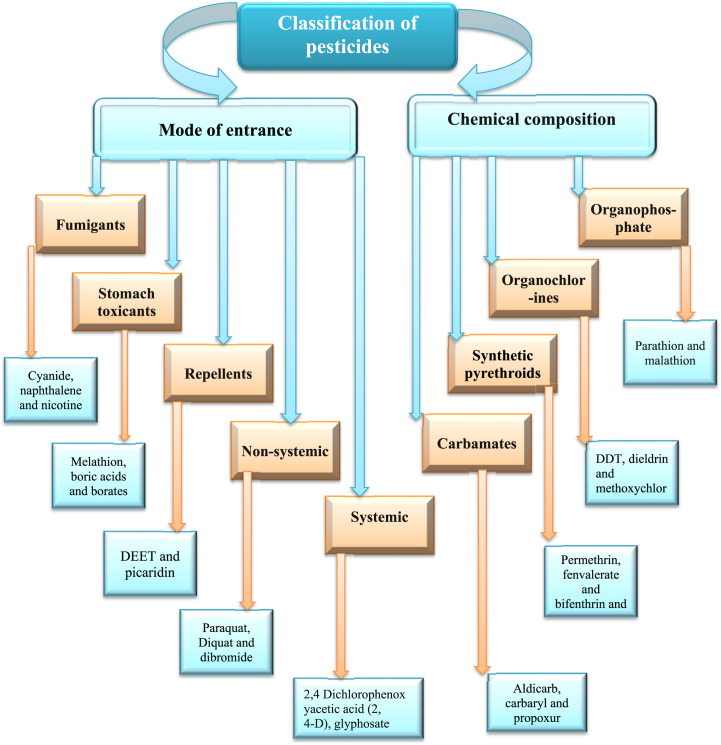
Pesticide classification on the basis of mode of entry and chemical composition.

Pesticides are classified using this method based on the target organism, and pesticides are given specific names to represent their actions. Pesticides are also divided into groups based on their intended use; for example, growth regulators encourage or inhibit the growth of pests, defoliants lead to the dropping of plant leaves, desiccants lead to the drying and killing of insects, repellents repel pests, and attractants fascinate and trap pests [51,52]. Some of the pesticides that are used in more than one category of pests may be included in more than one class of pesticide. Similarly, there are also several pesticides that control many insect classes and can be classified into multiple pesticide classes [53]. Aldicarb is extensively applied in Florida citrus production and may be categorized as an insecticide, acaricide, or nematicide as it controls insects, nematodes, and mites, respectively. Additionally, a common example is 2,4-D, which is applied as an herbicide for the control of broadleaf weeds. Repellents and attractants are considered pesticides since they are applied for pest control [29,45]. Furthermore, the classification of pesticides can be seen in Table 1, Table 2 and Fig. 1. It's necessary to remember that these categories are not exclusive, and many pesticides may appropriate into more than one category depending on their properties and intended uses. Furthermore, laws and classifications may differ between nations or areas, thus it's important to consult to regional laws and regulations for specific pesticide classifications.

**Table 1:** Pesticides classification on the basis of toxicity.

Types	Levels of toxicity	LD50 for the rat examples		Reference
		Oral	Dermal	
Class Ia	Extremely hazardous	<5	<50	[54]
Class Ib	Highly hazardous	5–50	50–200	
Class II	Moderately hazardous	50–2000	200–2000	
Class III	Slightly hazardous	>2000	>2000	
Class IV	Unlikely to present acute	5000 or higher

**Table 2:** Classification of pesticides on the basis of target pests.

Pesticides types	Function	Example of pesticides	References
Herbicides	Herbicides are chemical which are applied to control undesirable vegetation	Triazines, amides, urea derivatives, sulfonyl urea, uracil, carbamates herbicides, bipiridils and dinitroanilines	[29,[55], [56], [57], [58]]
Insecticides	Insecticides are chemicals used to kill or prevent insects**.**	Organophosphates, chlorinated, hydrocarbons, pyrethroids and carbamates	
Rodenticides	Rodenticides are pesticides that kill rodents.	Chlorophacinone and warfarin	
Fungicides and Bactericides	Fungicides and bactericides inhibit or mitigate the harm caused by fungi and bacteria	Benzimidazoles, diazines, morpholines, diazoles and triazole	
Acaricides	Kill mites that feed on animals and plants	Bifenazate	
Algaecides	Chemical substance which are used to kill and prevent the growth of algae	Copper sulfate	
Silvicides	Uses against the woody vegetation	Tebuthiuron and cacodylic acid	
Larvicides	Prevents the growth of larvae	Methoprene and temephos	
Ovicides	Substance that uses to kill particularly eggs	Benzoxazin	
Nematicides	Nematicides are chemicals used to kill nematodes and act as plants parasites	Aldicarb and carbamate	
Piscicides	Act against fishes	Rotenone, niclosamide and antimycin A	
Desiccants	Act on plants by drying their tissues	Boric acid	
Termiticides	Chemicals specifically designed to eliminate termites.	Fiproni and chlorantraniliprole

#### 3.1.3 Commercial pesticides formulations

The term “commercial formulations” refers to the range of product kinds wherein pesticides are designed to be secure, efficient, and easy to apply. While reducing potential dangers to people, pets, and the surroundings, these formulations are made to deliver the active components to the targeted pests [59,60]. Different types of commercial pesticide formulations are available in use that include liquid concentrates, wettable powders, soluble powders, dust, aerosol formulations, and microencapsulated [[61], [62], [63]]. Several criteria should be considered when selecting a commercial pesticide formulation in order to ensure efficient and secure pesticide use such as pest and crop, application method, persistence and residual effects, ensuring compliance with regional regulations and safety norms, and simplicity of handling are some significant elements to consider [64]. It is essential to carefully read and follow the manufacturer's directions for correct pesticide formulation handling, mixing, and application [65]. Various pesticides formulations can be seen in Table .3.

**Table 3:** Different commercial pesticides formulations.

Different pesticides formulations	Description	References
Granules	Pesticide-containing tiny pellets or granules make up granular formulations. Usually, they are dispersed across the target.	[[61], [62], [63],[65], [66], [67]]
Wettable Powders	These pesticide particles are finely powdered and combined with innocuous substances. Before applying, they are meant to be combined with water to create a suspension. They are renowned for their stability and extended shelf life.	
Soluble Powders	Soluble powders are like to wettable powders but are framed to dissolve completely in water.	
Liquid concentrates	Usually, these compositions are applied after being diluted with water. Frequently, they are emulsifiable concentrations.	
Dusts	They are usually applied by means of a dust applicator. Dust formulations are normally used in regions with low moisture or anywhere liquid uses are not possible	
Baits	Baits are formulated (gels, solid blocks, pastes) to attract insects like ants, cockroaches and rodents	
Aerosols:	Aerosol formulations are compressed containers that emit a fine spray in order to control flying insects	
Microencapsulated	Microencapsulated pesticides are little capsules that enclose the active component. They deliver controlled release and can adhere to surfaces for extended efficiency	
Baits	Baits are designed to draw pests and contain the active component insecticide. They are frequently used to control insects such as ants, cockroaches, and rodents. Baits can come as gels, pastes, or solid blocks	
Nano-based Pesticide Formulation	Through controlled release mechanisms, the development of nano-based pesticide formulation attempts to precisely deliver the adequate quantity of the active components

**Table 4:** Different herbicides and their mechanisms of action.

Herbicide	Mechanisms of action	Reference
Phosphinic acid	Glutamine synthase Inhibition	[[108], [109], [110], [111], [112], [113], [114], [115]]
Imidazolinone, Sulfonylurea, Triazolopyrimidine	Inhibit the acetohydroxyacid synthase	
Pyridazinone, Isoxazole, Pyrazole, Triketone, Triazole, Isoxazolidinone, Diphenylether	Inhibition of phytoene desaturase or 4- hydroxyphenylpyruvate dioxygenase	
Chloroacetamide, Oxyacetamide, Acetamide	Inhibit fatty acid synthase	
Benzoic acid, Pyridine carboxylic acid, Quinoline carboxylic acid	Stimulate the transport inhibitor response protein 1	
Diphenyl ether, thiadiazole, phenylpyrazole, oxadiazole, pyrimidinone, triazolinone	Protoporphyrinogen inhibition	
Phtalamate, semicarbazone	auxin transport inhibition	

##### 3.1.3.1 Commercial pesticides toxicology

Commercial pesticides include an extensive variety of chemicals with varied toxicity extents [68,69] (see Table 4). It is crucial to take into account that pesticide toxicity can be affected by a variety of factors, including the active components, formulation, quantity, and way of exposure [70]. Chlorpyrifos is an organophosphate insecticide that is used for controlling a number of pests [71]. It poses a moderate to serious risk to humans and has been connected to negative effects on neurodevelopment, particularly in young infants [72]. Because of safety issues, chlorpyrifos has been severely restricted in some countries [73,74]. Non-target creatures, such as fish, birds, and beneficial insects, can be severely hazardous to chlorpyrifos. It has the potential to pollute water bodies and exist in the environment [75]. Glyphosate is a broad-spectrum herbicide [76]. It has low acute toxic consequences such as, vomiting, nausea, abdominal pain, and diarrhea. According to certain research, glyphosate has been shown to act as an endocrine disruptor, altering hormonal systems [77,78]. Paraquat, another commercial herbicide, is used to suppress weeds in a variety of crops. It is extremely poisonous and can result in serious poisoning if consumed or applied to the skin [79]. One of the most noticeable and significant consequences of paraquat poisoning is its effect on the lungs. Inhalation or unintentional ingestion can cause pulmonary fibrosis, a condition described by the damaging and thickening of lung tissue. This can lead to irreversible lung damage and respiratory failure [80]. Due to its link with lung destruction, it is prohibited in a number of countries [81]. Malathion-containing products are additionally employed outdoors for controlling a broad range of insects in agricultural areas and around households, such as mosquito control [82]. It is regarded as moderately hazardous to humans and can produce dizziness and nausea. Various malathion formulations are linked to delayed neurotoxicity [83]. Organophosphate-induced delayed neuropathy is a disorder that can cause gradual weakness, numbness, and loss of coordination [84]. These are a few examples; many other commercial insecticides with differing degrees of toxicity are available [84,85].

Commercial pesticides used in agriculture and household settings contain a variety of co-formulants or adjuvants, and the toxicity of these compounds varies according to their particular composition and concentration. Co-formulants are added to pesticides to improve their stability, application, or efficacy [86,87]. The content and toxicity of adjuvants, also known as co-formulants, can differ greatly amongst commercial pesticides. The active compounds in pesticide formulations are dissolved or dispersed using co-formulants that are frequently utilized in combination with their potential toxicity [88]. Examples of these co-formulants are solvents, surfactants, preservatives, and emulsifiers. Some solvents, such as petroleum distillates, can be harmful, and have adverse effects on the skin and other body systems [89,90]. Surfactants are chemicals that help insecticides distribute, moisten, and penetrate more easily. It has been discovered that certain surfactants, such as alkylphenol ethoxylates (APEs) and nonylphenol ethoxylates (NPEs), are harmful to aquatic life and have endocrine-disrupting effects [[91], [92], [93], [94]]. In an analogous way, pesticide shelf life and stability are improved by stabilizers and preservatives. Examples include several organometallic compounds that can be harmful including formaldehyde, a recognized human carcinogen [[95], [96], [97]]. Additionally, in pesticide formulations, emulsifiers are utilized to stabilize the combination of water and oil based ingredients. It has been discovered that certain emulsifiers, such as ethoxylated tallow amines (ETAs), are hazardous to aquatic life [98]. However, it remains imperative to handle pesticides carefully according to label directions and take the necessary safety measures to reduce exposure to potentially hazardous co-formulants.

When compared to the active components alone, the pesticide co-formulant shows higher toxicities. Synergistic effects can result from co-formulants interacting with the active chemicals in a pesticide formulation [60,88]. This means that some co-formulants, such as piperonyl butoxide, which is frequently employed in insecticide formulations and works as a synergist, can increase the toxicity of the active chemicals in a formulation, making the pesticide formulation more potent [88,99]. Co-formulants have the potential to increase the bioavailability of active ingredients, which may result in higher toxicity when compared to the active component alone [78]. Furthermore, by decreasing surface tension and promoting spreading, co-formulants containing surfactant alkyl polyglucoside, may increase the penetration of active substances that might be toxic [100,101]. It is important to note that the toxicity of pesticide formulations and the effects of active ingredients and co-formulants should be evaluated by regulatory agencies before being made commercial. However, the evaluation methods and regulations can vary across different regions.

### 3.2 Mechanisms of action of different pesticides

Pesticides may exhibit numerous mechanisms of action based on the target pest. Here are a few typical mechanisms of action of pesticides.

#### 3.2.1 Herbicides

Amino acid synthesis inhibitors act on an enzyme to inhibit the production of certain amino acids, which are important for the development and growth of plants. One type of herbicide stops the enzyme acetolactate synthase (ALS), which makes branched-chain amino acids. ALS inhibitors include herbicides from the sulfonylurea family, and these compounds have a wide range of selectivity. There is a process by which plants make the aromatic ring amino acids phenylalanine, tryptophan, and tyrosine. The herbicide glyphosate has been made commercially available to stop that process. The enzyme 5-enolpyruvoyl-shikimate-3-phosphate synthase is blocked by the herbicide glyphosate and stops the growth of plants. Another enzyme that helps to make amino acids is glutamine synthetase, which makes glutamine from ammonia and glutamate. This enzyme is also a target for herbicides because it helps to make amino acids [[102], [103], [104]].

Some herbicides work by targeting the microtubule-forming protein tubulin, which is necessary for eukaryotic cells. These substances prevent regular cell division by attaching to tubulin. Herbicides that hinder cell division can be found in many different chemical classes, such as pyridines, benzoic acids, and dinitroanilines. Many herbicides work by preventing photosynthesis, which is a crucial mechanism for plants. Nitrogen-based substances, including as triazines, phenylureas, nitriles, pyridazines, and phenyl carbamates, are known to limit photosynthesis. Herbicides work in a variety of ways, including by preventing the production of free radicals, preventing photosynthesis, harming the electron transport system, and destroying protective pigments [[105], [106], [107]]. Herbicides can have a variety of modes of action, which determines how they affect plants. Mechanisms of action of different herbicides can be seen in Table 4.

#### 3.2.2 Insecticides

Numerous pesticides work by interfering with nervous system signals. Signal-interrupting chemicals are frequently potent toxins. Of this group, pyrethroids and organochlorines are the most important insecticides. Organochlorines, such as lindane and endosulfan, can indeed block gamma-aminobutyric acid (GABA) channels and interfere with chloride ion (Cl-) flux in the nervous system. GABA is an inhibitory neurotransmitter in the central nervous system that plays a crucial role in regulating neuronal excitability. It binds to GABA receptors, which are chloride ion channels, causing an influx of chloride ions into the neuron. This influx of negatively charged chloride ions hyperpolarizes the neuron, making it less likely to generate an action potential and reducing neuronal activity [[116], [117], [118]].

Furthermore, inhibitors of cholinesterase and chitin synthesis exhibit an important role as insecticides. Organophosphorus insecticides phosphorylate the esteratic active site of the acetylcholinesterase (AChE), AChE is responsible for breaking down acetylcholine, a neurotransmitter that is involved in transmitting nerve impulses [119]. While carbamates like organophosphorus insecticides, also inhibit the enzyme AChE, both of them produce their insecticidal action through cholinesterase inhibitors. Polysaccharides like chitin are found all over the world. Chitin is found in arthropods and fungi but not in plants or mammals. Benzoylureas distress the synthesis of chitin in insect cuticles by disrupting the connection of N-acetylglucosamine units to the chitin chain and inhibiting the molting process of insects [120,121]. Mechanisms of action of different insecticides can be seen in Table 5.

#### 3.2.3 Fungicides

Fungicides act also through different modes of action that include inhibition of cell division, inhibition of ergosterol synthesis and by acting on sulfhydryl groups of enzymes of fungal cells (Table 6.) (see Table 5). Sulfhydryl (SH) are found in many enzymes that exhibit a significant role in fungicidal action. Dithiocarbamate fungicides attack the fungal cells enzymes and coenzymes that have SH group. Pesticides like captan and folpet work with enzymes that have SH groups. These fungicides change the structure and function of the cell membranes and stop the enzyme system [[129], [130], [131], [132], [133]].

**Table 5:** Different insecticides and their mechanisms of action.

Insecticide family	Mechanisms of action	Reference
Carbamates	Acetylcholinesterase Inhibitors	[[122], [123], [124], [125], [126], [127], [128]]
Organophosphates	Inhibiting the activity of an enzyme called acetylcholinesterase	
pyrethroids	Targeting the nervous system of insects, specifically by interacting with sodium channels	
Hexathiazox	Mite growth inhibitors	
Neonicotinoids	Specifically bind and interact through the insect nicotinic acetylcholine receptor site	
Diafenthiuron, Propargite, Tetradifon,	ATP synthase inhibitor	
Buprofezin, Benzoylureas	Chitin synthesis inhibitor	
Diamides	Interacting with a specific receptor called the ryanodine receptor	
Rotenone	Interfering with the electron transport chain within complex I in mitochondria	
Oxadiazines	Voltage gated sodium channel blocker

**Table 6:** Different fungicides and their mechanisms of action.

Fungicides	Mechanisms of action	Reference
Dichlormate, pyriclor, amitrole,	Inhibition of carotenoid synthesis	[108,[135], [136], [137], [138], [139], [140]]
Triazole fungicides (e.g., tebuconazole, propiconazole,	Inhibit the biosynthesis of ergosterol	
Benzimidazoles	Inhibits fungal cell division by interfering with microtubule	
Azoxystrobin	inhibits the respiration process in fungi by binding to a protein complex	
Chlorothalonil	disrupting enzyme activity, and interfering with various metabolic processes	
Carboxamide derivatives	Succinate dehydrogenase inhibitors	
Ziram	Inhibition of Metalloenzymes	

Tubulin is an essential protein of the intracellular skeleton, and benzimidazole inhibits the building of the intracellular skeleton by reacting with tubulin. Generally, pesticides prevent cell division by inhibiting the formation of microtubules. There are a lot of different types of fungicides that work this way; these include thiabendazole, carbendazim, and benomyl [134].

**Fig. 2:**
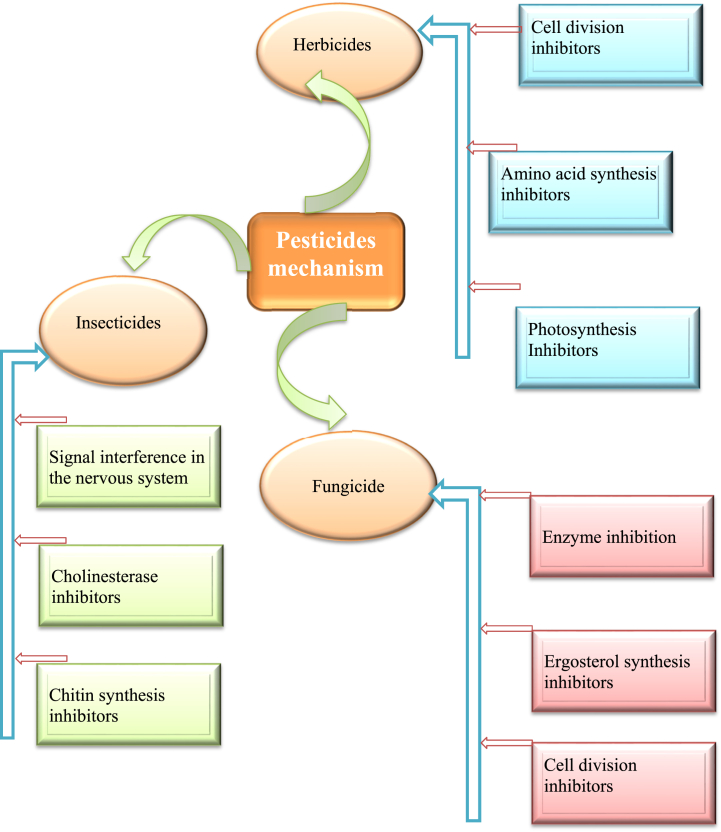
Various mechanisms of action exhibited by different pesticides.

Ergosterol-inhibitor fungicides can kill many different types of fungi, they stop the higher plants from making sterols and gibberellins. Sterols synthesis is a complex process, fungicides act on the synthesis path. There are several fungicides that are used as demethylase inhibitors such as pyridines, morpholines and piperazines [141]. Furthermore, several fungicides that target many sites of the fungi that include inhibition antioxidant enzymes that lead to interrupt cell redox balance and several of them also prevent the nuclear factor-kB signaling cascade consequently distress the numerous biochemical actions [142]. Mechanisms of different pesticides has been depicted in Fig. 2.

### 3.3 Effects of pesticides on human health

#### 3.3.1 Neurological disorders

Several studies have been carried out to determine the effects of pesticides on neurological illnesses. Alzheimer's disease and Parkinson's disease are the most common disorders connected to pesticide neurotoxicity [143,144].

##### 3.3.1.1 Alzheimer's disease

Alzheimer's disease (AD) is the utmost frequent form of dementia among the elderly, and it's becoming more widespread around the world [145]. Pesticide exposure has been linked to neurological problems in several studies. Due to differences in their job activities, men have been found to be exposed to more pesticides than women [146]. A study of Cache County agricultural community reported that pesticide exposure (organochlorines) increases the threat of AD and dementia. Evidence suggests that exposure to pesticides for a long period of time can cause brain damage and lead to the progression of AD. In a meta-analysis, a direct link between pesticide contact and AD was discovered, confirming the idea that pesticide contact is a risk factor for AD [30,147]. Case-control studies were conducted to investigate pesticide exposure linked to Alzheimer's disease and their controls. The findings of the first investigation revealed that pesticides pose a risk. A second study found a link between elevated DDE levels in the blood and AD [148,149]. In the case of neurodegenerative illnesses, it has been documented that prenatal exposure to organophosphates causes changes in schoolchildren's mental function [150,151]. An additional study reported that children exposed to pesticides (organochlorines) through prenatal and postnatal exposure develop cognitive and autistic syndromes. Pesticide exposure damages the basal forebrain cholinergic neurons that lead to memory and sensory problems. During the Gulf War in 1991, US armed troops exposed to sarin and cyclosarin developed neurological problems [152,153]. Due to a Japanese terrorist strike in 1995, Tokyo was exposed to sarin gas. Although some victims died and others healed, mental impairments lasted for years. Many productive lives are lost as a result of these chronic mental health disorders caused by accidental or suicidal organophosphate poisoning [154].

##### 3.3.1.2 Parkinson's disease

Parkinson's disease (PD) is another common neurological disease after AD. Pesticides and their metabolites disrupt mitochondrial function and modify xenobiotic metabolism, resulting in Parkinson's disease (PD) [14,155,156]. In another study, it was shown that rats exposed to rotenone lead to neurodegeneration in the peripheral nervous system over time as well as a reduced conduction velocity of motor nerves, particularly in the sciatic nerves. It is caused by a lack of dopamine and a chemical synapse breakdown in the peripheral nervous system [157]. There is a link between exposure and Parkinson's disease. When combined with the preceding meta-analysis, 15 out of 26 studies found a link between Parkinson's disease and pesticide exposure. It has been confirmed from cohort and case-control studies that pesticide exposure increases the risk of PD, more specifically mancozeb and paraquat [158]. These findings reveal a strong link between Parkinson's disease and pesticide exposure [159].

The connection between paraquat and PD as a potent inducer of oxidative damage, which triggers reactive oxygen species (ROS) production [160]. PD is a multifactorial ailment comprising numerous biochemical pathways, for instance mitochondrial dysfunction, oxidative injury, ER stress, alteration in dopamine catabolism, inactivation of tyrosine hydroxylase, and reduction in brain-derived neurotrophic factor (BDNF), finally resulting in apoptosis of the dopaminergic neurons in the substantia nigra pars compacta [161,162]. The utilization of paraquat-based in vitro and in vivo studies, which either directly or indirectly contribute to the progression of the disease under the aggravating state of oxidative damage [163]. Primary mechanism of oxidative damage leading to the death of neurons that generate dopamine is the generation of ROS and reactive nitrogen species (RNS) by paraquat. According to epidemiological research, those who are exposed to paraquat over an extended period of time may be more likely to acquire PD [164]. Systematic review and meta-analysis exhibited the herbicide paraquat linked with the progress of PD. Nine case-control studies' findings showed that paraquat exposure increased the risk of PD by 25%. The sole cohort study present showed an OR of 1.08 that was not significant. Results from subgroup analysis also showed that participants who were exposing paraquat for a longer duration of time or those who were co-exposed to paraquat and any other dithiocarbamate had higher PD frequency [165]. A probable relationship between occupational pesticide exposure and PD death was discovered in cohort research in the Netherlands that involved 62,573 women and 58,279 men between the ages of 55–69 years [166]. Furthermore, persistent exposure to heavy metals and pesticides also leads to the onset of Parkinson's disease at an earlier age. Furthermore, they discovered that the length of exposure was a key element in determining the degree of such effects [31,167].

##### 3.3.1.3 Neurotoxic effect

Many organophosphate and carbamate insecticides work by inhibiting the enzyme acetylcholinesterase, which is responsible for breaking down the neurotransmitter acetylcholine. By inhibiting AChE, these pesticides lead to an accumulation of acetylcholine, resulting in overstimulation of cholinergic receptors in the nervous system. This can cause symptoms such as excessive salivation [168,169]. Certain pesticides, such as organochlorines like indane and endosulfan, may react with the central nervous system's GABA receptors. They have the ability to modify GABA receptor activity, which can change inhibitory neurotransmission. This disturbance of GABAergic signaling can cause increased lipid peroxidation and damage to DNA in brain tissue, as well as neuronal hyperexcitability, seizures, and other neurological symptoms [170], estradiol, which causes disorders of anxiety [171] and dicofol, which has consequences on sensory, motor, or cognitive functions [172,173]. It has been demonstrated that certain pesticides, including pyrethroids and organophosphates, induce oxidative stress in the neurological system. They lead to an oxidant-antioxidant imbalance by generating ROS and interfering with antioxidant defense mechanisms. Oxidative stress can cause damage to neurons, change the way cells function, and quicken the onset of neurodegenerative diseases [[174], [175], [176]].

##### 3.3.1.4 Cognitive effects

Despite rising evidence that exposure to pesticides causes neurological illnesses and neurobehavioral effects, data from epidemiological studies of chronic pesticide exposure is scarce [177]. In the prospective Investigation of the Vasculature in Uppsala Seniors (PIVUS), three organochlorine (OC) pesticides, including *trans*-nonachlor, hexachlorobenzene, and p,p′-DDE plasma concentrations, were tested in 989 men and women aged 70 years. According to the results, it was found that the OC with a higher concentration of plasma had three times greater future cognitive impairment risk in comparison to lesser OC levels [178]. An additional study looked at the pesticides impact on neurobehavioral function in 929 French vineyard personnel aged 42–57 years old; as a result, the chance of failing a cognitive assessment was greater in exposed personnel, and odds ratios ranged from 1.35 to 5.60 [179]. Three separate investigations on the effects of prenatal OP exposure on children's cognitive capacities concluded lower IQ, perceptual reasoning, and poorer memory [[180], [181], [182]].

#### 3.3.2 Cancer

Cancer is one of the most widely prevalent diseases across the world [183,184]. Direct contact with pesticides is the leading cause of cancer around the world. This is a global issue that is presently fascinating researchers from all around the world [185,186].

##### 3.3.2.1 Breast cancer

Pesticide exposure and its effects on health are prime considerations. There is rising scientific confirmation that chemical exposure, particularly pesticides, is linked with an increased occurrence of breast cancer [11]. It has been reported that pesticides produce carcinogenic effects by disrupting estrogen receptors or damaging DNA in breast tissue and boosting malignancy and DNA mutation in vulnerable individuals. Pesticides employed in modern agriculture are suspected of having a negative impact on human reproductive health, particularly breast and colon cancer, through endocrine disruption mechanisms [[187], [188], [189]]. Chlorpyrifos (CPF) in pesticides was shown to cause a redox imbalance in breast cancer cells, altering the antioxidant defense system [190]. Individually, several OCs have been associated with breast cancer due to ability to produce oestrogenic consequences on mammary cells. Dichlorodiphenyltrichloroethane (DDT), chlordane, heptachlor epoxide and heptachlor were the most common pesticides discovered in milk samples in pesticide studies. Women with more serum concentrations of DDE, the primary DDT metabolite, had a greater risk of breast cancer than women with low concentrations [191,192]. Cohn et al. studied a group of females in Oakland, California, in a nested case-control study and they discovered that higher blood p,p'-DDT concentrations were linked to a higher risk of breast cancer, but only in women who were exposed before the age of 14 [193]. Furthermore, a probable link between blood concentrations of organochlorine insecticides, polychlorinated biphenyls, and xenoestrogens effects was observed in a Tunisian female population, and a risk of breast cancer was discovered [194].

##### 3.3.2.2 Bladder and colon cancer

Heterocyclic aromatic amines have been discovered in a number of cases of colon and bladder cancer [195]. The risk of cancer is proportional to the level of exposure and the length of time spent in the environment [196]. Imidazolinone herbicides, including imazaquin and imazethapyr, exhibited their role in bladder cancer, as reported by a prospective cohort study involving pesticide applicators (57,310) in the United States [197]. Imidazolinone herbicides including imazaquin and imazethapyr exhibited their role in bladder cancer, it was reported by a prospective cohort study involving pesticide applicators (57,310) in the United States [198]. Further, in an Egypt case-control study conducted on male agricultural workers with 881 controls and 953 cases, it was found that exposure to pesticides exhibited bladder cancer risk with an OR of 1.68 and a 95 percent confidence interval (CI) of 1.23–2.29% [199].

##### 3.3.2.3 Brain cancer

The precise mechanisms through which pesticides may contribute to the formation of brain tumors are unknown. Some pesticides have been demonstrated to have genotoxic effects, which means they can harm DNA and potentially contribute to cancer cell development [200]. Pesticides also exhibit the ability to impair hormonal balance and so contribute to cancer development [201]. Greenop et al. (2013) found that preconception pesticide exposure, and likely exposure throughout pregnancy, is linked to an elevated incidence of infantile brain tumors. It is possible that both parents should avoid pesticide exposure during this period [202]. Additionally, a related study found a strong correlation between residential pesticide exposure and children brain tumors, namely indoor pesticide exposure associated with gliomas. The data linking pesticide exposure to juvenile brain cancer is supported by parental occupational exposure to pesticides [203]. Scientific research is being done on the connection between pesticide exposure and brain cancer, namely meningioma and other forms of brain tumors [204,205]. There is currently limited and inconsistent data to support the hypothesis that pesticide exposure may raise the incidence of brain cancer, despite some studies suggestions to that effect.

##### 3.3.2.4 Liver cancer

Liver cancer risk may rise with extended exposure to several pesticides, especially those categorized as carcinogens [206]. Scientific study and inquiry are being conducted on the connection between pesticides and liver cancer [207]. Certain pesticides have been linked to a higher risk of liver cancer, while their specific mechanisms are still unclear [207]. It has been shown that certain pesticides, such as some fungicides and herbicides, have genotoxic qualities, which means they might harm DNA and perhaps cause cancerous alterations in liver cells. Possible pathways that may link pesticide exposure to cancer include inflammation, oxidative stress, and disruption of hormone control [201,[208], [209], [210]]. Organochlorine pesticides are among the particular pesticides that have been linked to liver cancer [211]. Over time, pesticides have been shown to build up in the body and have been linked to cancer. While pesticide exposure by itself might not be enough to cause liver cancer, it can raise the risk when combined with other variables. The risk of liver cancer has been researched in relation to occupational exposure to specific pesticides [212]. Employees in agriculture, especially those handling and applying pesticides, may be more exposed [213]. More study is needed to demonstrate a definite cause-and-effect relationship, even though studies have shown associations between occupational pesticide exposure and an elevated risk of liver cancer.

#### 3.3.3 Effect on reproduction

##### 3.3.3.1 Fertility

Fertility refers to a woman's or a man's capacity to become pregnant within a year, and it encompasses both male and female aspects such as sperm quality and infertility. Several studies showed no link between pesticide exposure and sperm abnormalities; others found no link between pesticide exposure and sperm abnormalities [17,[214], [215], [216], [217]]. Discovered a link between organophosphate metabolites and sperm sex aneuploidies, while another study discovered a link between erectile dysfunction and pesticide exposure [218]. Oliva et al. (2002) in one study, women who worked with herbicides in the two years before trying to conceive had a higher chance of infertility [215,218].

Vegetable consumption has been linked to pyrethroid pesticide metabolite levels in the urine in previous research. Men attending a reproductive clinic had reduced total ejaculate volume, sperm count, and proportion of morphologically usual sperm when they ate fruits and vegetables with significant pesticide residues [219,220]. Intake of low-to-moderate pesticide residue was linked to sperm morphology. These data demonstrate that dietary pesticide exposure used in farming might be enough to alter human spermatogenesis [221].

##### 3.3.3.2 Birth defects

The most frequent and devastating fetal congenital disorders are neural tube defects (NTDs). OCPs (organochlorine pesticides) are widely used in the environment [222]. In a rural area of northern China, 119 women with NTD-affected pregnancies and 119 females who had healthy neonates as controls were selected for this study. To study the link between in utero exposure to OCPs and NTD risk, researchers used OCPs concentrations in umbilical cord tissue as markers of prenatal exposure. GCMS was employed to determine the concentrations of 20 OCPs, and 16 of the 20 OCPs involved in the studies Individual odds ratios and 95 percent confidence intervals (95 percent CIs) for the correlations between NTD risk and levels of individual OCPs were calculated [223].

Pesticides and birth abnormalities were investigated in fifteen studies from nine nations, ranging from 63 to 77. Pesticide exposure was consistently associated with an elevated risk of different complications that include urogenital anomalies, limb disorders, orofacial clefts, and ocular anomalies as specific problems [[224], [225], [226]]. Pesticide exposure by parents also enhanced the risk of any birth abnormality [[226], [227], [228]].

##### 3.3.3.3 Fetal death

Natural abortion, stillbirth, fetal death, and neonatal death are examples of pesticide-exposed effects [229]. The findings were similar across multiple study designs, with positive correlations with pesticide exposure found in 9 of 11 trials [15,226,230]. The findings of the Ontario Farm Study pointed to critical times when pesticide exposure is most dangerous. Early first-trimester abortions were linked to preconception exposure, while late spontaneous abortions were linked to postconception exposure [231]. In research from the Philippines, the probability of spontaneous abortion was six times higher in farming households that used a lot of pesticides compared to those that used integrated pest management [230].

#### 3.3.4 Respiratory disorders

Recurrent attacks of bronchial constriction produce dyspnea, wheezing, and coughing, making it a significant life-threatening condition of the lungs. Increased bronchial hyper responsiveness raises the risk of getting asthma. Pesticides and asthma are closely linked, as exposure to certain pesticides can increase the risk of developing asthma or exacerbate existing asthma symptoms. Several studies have found associations between pesticide exposure and the development of asthma, particularly in children. Pesticides used in agriculture, such as organophosphates and pyrethroids, have been linked to an increased risk of asthma in children living near agricultural areas [232,233]. Pesticides produce asthma symptoms that include edema, irritation, endocrine disruption, and immunological suppression [232,[234], [235], [236]]. Evidence confirms that exposure to agricultural pesticides is linked to higher rates of lung cancer, particularly when exposure persists for more than 2 days per month [237].

The bronchial mucosa is immediately damaged by pesticides, which makes the airway extremely sensitive to allergens. For occupational, household, and environmental exposures, pesticides including OC, paraquat, carbamate, OP, and pyrethroid exhibited the highest connection with asthma [238]. Nevertheless, the majority of pesticides are weakly immunogenic, while a few are strong enough to harm the bronchial mucosa. Pesticide use in farms was found to be related to atopic asthma (OR = 1.46; 95 percent CI: 1.14–1.87) in research involving 25,814 farm women in the United States [57,239]. Chemicals can affect respiratory health in a variety of ways. For example, polycyclic aromatic hydrocarbons (PAHs) linked with small particles move in the lungs, producing swelling and impairing the respiratory system. According to epidemiological studies, there is a link between PAH exposure and air pollution concentrations and the development of allergic and non-allergic asthma, increased asthma symptoms, the risk of asthma exacerbations, and a decrease in lung function, but there is a low level of evidence based on current data. The strongest evidence for a link between asthma development and lung function in children is presented [[240], [241], [242]]. Immunoglobulin E (IgE), inflammation, mast cells, oxidative stress, and epithelial as well as endothelial dysfunction probably play a role in these pathophysiological developments [243,244]. While the dose and duration of PAH exposure are linked to the risk of asthma. Diisocyanates cause particular sensitivity, which is linked to respiratory problems. Furthermore, skin sensitization can occur as a result of diisocyanate exposure. Even a small amount of diisocyanate exposure can cause sensitivity and asthma. IgE-mediated asthma is sometimes seen in isocyanate-induced asthma, however, particular sensitization is frequently seen in bronchial examinations without specific IgE [232,245]. The sensitive group for asthma has been listed in Table 7.

**Table 7:** Pesticides and other substances with a sensitive group and an exposed association with asthma.

Pesticides and other chemicals	Sensitive group	Association of asthma	References
Polycyclic aromatic hydrocarbons (PAHs)	Children and subjects with allergies	Yes	[[240], [241], [242]]
per-and polyfluoroalkyl substances (PFAS)	children, fetuses and pregnant women	Probably	[[246], [247], [248]]
Diisocyanates	Subjects working with diisocyanates.	Yes	[245,249,250]
Pesticides	Children early in life, fetuses and pregnant women	Yes	[251]
Cadmium (Cd)	Postmenopausal and pregnant women, and children in the postnatal period and toddlers	No	[252]
Arsenic (As)	Children	No	[232,252]
*p*-phenylenediamine (*p*-PDA)	Occupationally exposed subjects to *p*-PDA	Probably	[249,253]
Cr(VI)	Occupationally exposed subjects to Cr(VI)	Yes	[254]
Phthalates	Young children		[255,256]
Mercury (Hg)	Fetuses, newborns, young children, and people who eat a lot of sea foods	No	[252,256]

#### 3.3.5 Non-alcoholic fatty liver disease (NAFLD)

The condition known as non-alcoholic fatty liver disease (NAFLD) is defined by the buildup of extra fat in the liver and is not connected to heavy alcohol use [257,258]. Although obesity, insulin resistance, and metabolic syndrome are the main risk factors for NAFLD [259,260], there is some evidence to suggest that some pesticides may also play a role in the onset or progression of NAFLD [260]. It is unclear how exactly pesticides may cause NAFLD. Nonetheless, it is believed that exposure to pesticides might affect the composition of the gut microbiota, induce oxidative stress and inflammation, and interfere with lipid metabolism, all of which are linked to the onset and advancement of NAFLD [[261], [262], [263]]. Furthermore, pesticide metabolism can produce reactive oxygen species, resulting in oxidative stress and liver damage [264]. Pesticides may encourage the function of responsible enzymes for detoxification in the liver, such as cytochrome P450 enzymes [265]. Whereas this induction is a part of the liver's defense system, too much induction might result in the formation of reactive chemicals that can harm liver cells. NAFLD has been connected to a number of chemicals including polychlorinated biphenyls [266]. These compounds possess the capacity to induce inflammation, disrupt the hepatic lipid metabolism, and accelerate the fatty liver progression [[266], [267], [268]]. The development of NAFLD may also be influenced by pesticide residues from meals. Eating pesticide-contaminated food items, especially those heavy in fat, might increase one's exposure to pesticides overall and may have an adverse effect on liver health [262]. It is advised to prevent exposure to pesticide residues in food, maintain a balanced diet with an emphasis on organic produce where possible, and abide by safety precautions while using pesticides in residential or agricultural areas in order to lower the potential dangers associated with pesticide exposure.

#### 3.3.6 Diabetes

Exposure to environmental toxins appears to affect diabetes, according to new scientific data [269]. A number of studies have shown that certain pesticides may raise the risk of diabetes or impair glycemic control [270,271]. According to certain research, chronic exposure to some pesticides, such as organophosphates and organochlorines, may interfere with the endocrine system's normal operation, which can lead to the onset of diabetes. These insecticides may disrupt glucose metabolism and insulin signaling, resulting in insulin resistance and poor glucose control [272]. Pesticide exposure, mainly organochlorines (OC) and metabolites, is thought to increase the incidence of type 2 diabetes (T2DM) and associated complications [273]. Through the various studies, it has been found that there is a link between diabetes and serum concentrations of various pesticides that include dibenzofurans, polychlorinated dibenzodioxins, PCBs, and various organochlorine pesticides, including DDE, DDT, *trans*-nonachlor, oxychlordane, and hexachlorocyclohexane. Because most research was cross-sectional, the actual datasets had significant limitations. Only a few studies looked at selection bias and the confounding effect, and the majority of estimates used extremely broad confidence ranges. Exposure to organochlorines, for instance, polychlorinated biphenyls (PCBs) and p,p′-DDE, increases the risk of T2DM, according to a meta-analysis of 23 relevant publications [274]. In the USA, according to AHS data, a total of 506 (4.5%) women who worked with farming pesticides were diagnosed with gestational diabetes mellitus (GDM) during pregnancy [275]. Furthermore, five pesticides, including the organophosphates fonofos, parathion, and phorate, the herbicide 2,4,5-T/2,4,5-TP, and the organochlorine dieldrin, were found to be linked with diabetes [276]. Furthermore, a cross-sectional study was conducted with 92 non-exposed controls and 116 pesticide sprinklers in Bolivia, and it was discovered that the sprayer individuals had an atypical glucose control of 6.1 percent, compared to 7.9 percent for non-exposed people [277]. Moreover, elevated levels of heptachlor epoxide and DDT in human blood have been linked to the development of diabetic nephropathy [278,279]. A case-controlled study conducted in Bang Rakam suggested pesticide exposure is linked to diabetes [280]. Imazamox is a systemic herbicide that moves through the tissue of plants and inhibits the plants from generating necessary enzymes. In a study of imazamox, it was concluded that exposure to this herbicide leads to reduced β-islet cell size and increases the concentration of glucose and calcium [281]. Confirmations from experimental studies have been done on pesticides such as DDE, which are endocrine-disrupting chemicals that may lead to the progression of diabetes [282,283]. It is important to remember that diabetes is a complicated illness with numerous risk factors, such as genetics, lifestyle choices, and weight. One possible contributing cause among many is exposure to pesticides. For a complete understanding of the connection between pesticides and diabetes, more research must be conducted [284,285].

#### 3.3.7 Allergic reactions

The body develops a repelling response after the initial exposure to pesticides, but later exposure leads to an allergic reaction. Sensitization is the term for this process, and sensitizers are chemicals that trigger allergic reactions. Life-threatening shock, asthma, skin irritation like blisters, rash, rhinitis, open sores, and irritation of the nose and eyes like watery eyes, itching, and sneezing are a few examples of allergies. Unfortunately, determining which person may be allergic to which chemical is challenging [286,287]. Epidemiological data have revealed a potential link between asthma and phthalate exposure, and it was found that phthalates may aggravate pre-existing respiratory problems [255,288]. Home exposure to phthalates has been linked to allergies in children. High-molecular-weight phthalates may produce allergic symptoms in adults, according to a survey conducted in the United States [289,290]. Adults may develop asthma after being exposed to heated PVC vapors. Several phthalates have been shown to affect the mouse immune reaction to co-allergens and function as adjuvants in allergic responses in vitro [255,288,291]. The derivative *p*-Phenylenediamine (*p*-PDA) is a frequent contact allergen that causes skin sensitization, and its exposure may be linked to an increased threat of rhinitis and occupational asthma [249,253,292].

#### 3.3.8 Leukemia

**Fig. 3:**
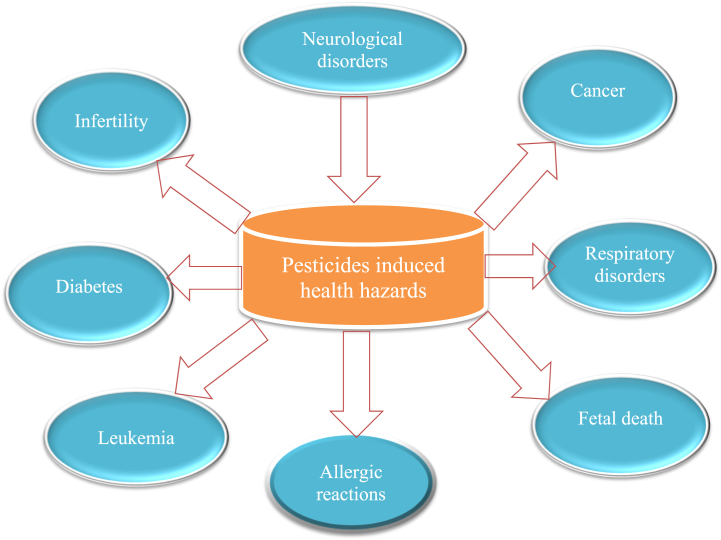
Various impact of pesticides on human health.

Pesticide exposure has been associated to an increased chance of getting leukemia, which is characterized by abnormal white blood cell production that can impair the body's capacity to fight infections and other disorders. Several studies have found a link between pesticide exposure and leukemia development [293]. Farmers, farmworkers, and pesticide applicators, who are often exposed to pesticides, have been found to have a higher chance of having leukemia than the general population [294]. Pesticide exposure is one of the leading reasons for acute leukemia. The impact of exposure to pesticides on childhood leukemia has been studied in the past. It was found that the odd ratios for acute lymphoblastic leukemia for 3 forms of exposure—just earlier conception, throughout gestation, and later birth—were 1.39, 1.43, and 1.36, respectively, in 12 case-control studies of pediatric leukemia. Several studies have found that parental pesticide exposure raises the incidence of pediatric leukemia by three times. A case-control study conducted in Iran on occupational farmers found that they had a considerably higher chance of acquiring acute leukemia than other workers, particularly their children, due to pesticide exposure [295]. According to the Children's Cancer Study Group, parental pesticide exposure is the primary cause of acute non-lymphoblastic leukemia, and children who are consistently exposed to household pesticides have a 3.5 times greater risk of leukemia development [296]. Pesticides are also a source of leukemia in infants born to exposed mothers during pregnancy; small children under the age of one have a seven-fold increased risk of developing leukemia if exposed to permethrin pesticide [12]. Various effects can be seen in Fig. 3.

According to certain research, there is a direct correlation between exposure to glyphosate and the incidence of non-Hodgkin lymphoma (NHL). Glyphosate was categorized as a “probable human carcinogen” by the International Agency for Research on Cancer (IARC), a specialized agency of the World Health Organization (WHO), in 2015 [297]. This classification was made based on limited data from human research and significant data from studies on animals demonstrating a link between glyphosate interaction and the development of NHL [[297], [298], [299]]. Glyphosate could disrupt the endocrine system by interacting with hormones involved in immunological function, according to one probable mechanism. This disruption could potentially raise the chance of developing NHL [300]. Another theory is that glyphosate might function as a genotoxic agent, which means it might harm DNA and perhaps cause cancer. According to some research, glyphosate may harm DNA, which may have a role in the onset of NHL [297].

### 3.4 Pesticides detrimental effects on the environment

Pesticides are well-known weapons for defending crops, ensuring high levels of harvest, and intervening in disaster conditions to eliminate parasitic infections. While pesticides have been recognized as a severe threat to the health and environment, they can easily migrate into other environmental compartments owing to runoff and leaching [[301], [302], [303]]. Widespread use and subsequent pesticide disposal by agriculturalists, the general public, and institutions offer abundant possible bases for spreading pesticide exposures in the environment. The sources of pesticide spread are broad and include dissolution in water, air, and soil, so it is quite difficult to totally control the spread and exposure of these health-hazard agents [304]. Pesticides can persist in the soil for extended periods, especially those with long half-lives. Continuous and excessive use of pesticides can lead to the accumulation of toxic chemicals in the soil, which can harm beneficial organisms such as earthworms and soil microorganisms. This can disrupt soil fertility and the overall health of the soil ecosystem [305,306]. When pesticides are sprayed in farming, they may travel in the air and spread to other areas of the environment, for instance, water and soil. Pesticides that are sprayed on the soil may be swept away and reach neighboring water bodies through surface runoff or may percolate down to soil layers and finally into groundwater. Pesticides exhibit a wide range of effects on the environment, from slight disruptions in the ecosystem to the destruction of species. Pesticides produce everything from acute fatalities to long-term side effects [302].

In studies conducted in Europe on 76 pesticides, residues were discovered in top soils across continent. One or more residues were discovered in 83 percent of the soils, while 58 percent of the samples included two or more residues. The greatest concentrations of glyphosate and its metabolites were obtained regularly. Numerous pesticides have been discovered in rivers, lakes, and surface water across Europe that could pose a potential harm to aquatic creatures [307,308]. Pesticide contamination in water poses the greatest threat to aquatic bodies, mostly through diminishing dissolved oxygen levels. They have an impact on aquatic animals at all stages of the trophic chain, from algae to fish. Pesticides have been detected in water and on surfaces; the widespread consumption of pesticides may lead to a decrease in the number of fish [309,310]. Pesticides are consumed by aquatic animals in different ways, including dermally, through inhalation, and through ingestion. Because herbicides kill aquatic vegetation, the oxygen content in the water drops rapidly, resulting in fish suffocation and decreased fish output. Aquatic species reproductive abilities were also harmed as a result of herbicide use near weedy fish nurseries [309]. Other studies have found that pesticides interact with soil microflora, microfauna, and macrofauna through altering soil chemistry and the interaction of soil chemicals with plant roots, as well as inhibiting processes such as rhizo-biological bacteria's fixation of atmospheric nitrogen [311].

Organ chlorine levels higher than the US EPA threshold were found in 58 percent of drinking water samples gathered from various hand pumps and wells around Bhopal, according to an Indian survey. In India, at least one pesticide was found in over 90% of aquatic and fish samples from each stream. In drinking water analysis across India, pesticides and their metabolites were detected in high concentrations, including DDT metabolites such as hexachlorocyclohexane and endosulfan [[312], [313], [314]].

Pesticides significantly affect the biodiversity of both animals and plants, in addition to non-target organisms. In the food chain, pesticides can build up and affect wildlife indirectly. Prey that is contaminated with pesticides can expose predatory species at the top of the food chain, such as raptors and mammals, to high levels of the chemicals. This may result in decreases fertility, weakened immune systems, and a decline in populations [315,316]. People with weakened immune systems are generally more vulnerable to various infections like bronchitis, pneumonia, and influenza [317,318]. Pesticides can contaminate natural ecosystems in two ways, depending on their solubility. Pesticides that are dissolved in water can end up in groundwater, rivers, lakes, and streams, causing harm to non-target animals. Bioamplification disrupts the whole ecosystem, and species at higher trophic levels will perish as a result of increased toxicity in their bodies. As a result, the number of secondary consumers has increased while the number of primary consumers has declined [319].

**Fig. 4:**
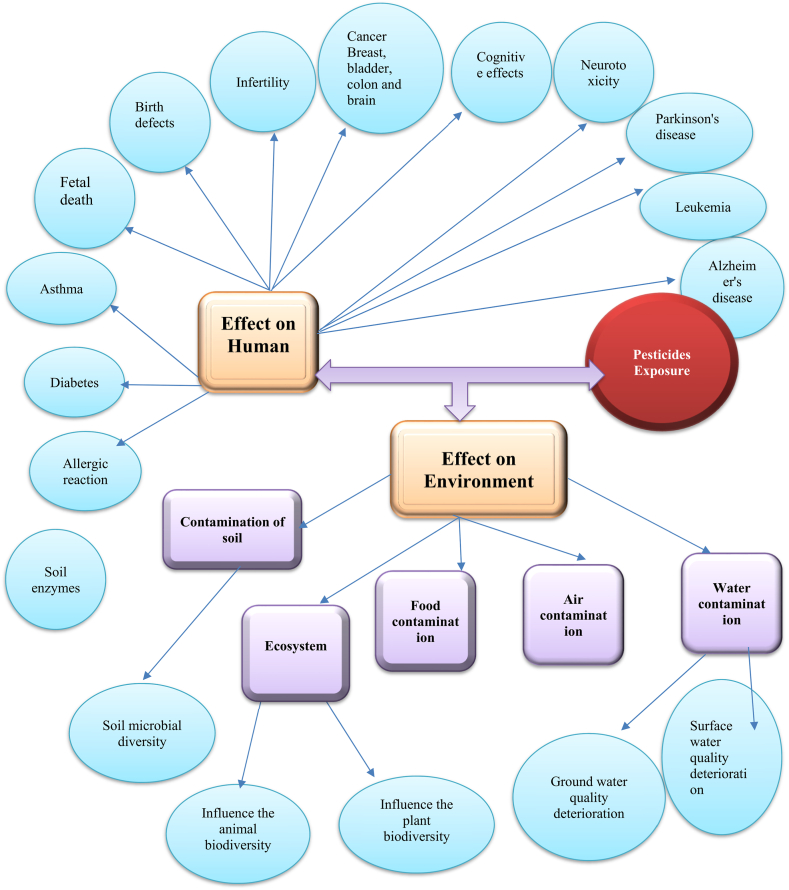
Pesticides effects on human and environment.

Pesticide resistance can develop in targeted pests after prolonged and intensive application. When pests develop resistance to a specific pesticide greater doses or more toxic pesticides may be required to achieve the desired effect. This can lead to a cycle of higher pesticide usage, exacerbating the negative environmental effects [320,321]. Pesticides effects on human and environment has been depicted in Fig. 4.

### 3.5 Approaches for safety measures

In the current pesticide-induced environment, health hazards and safety concerns are critical issues. There are several regulatory bodies that deal with pesticide safety. Agricultural scientists began developing alternative crop management systems to decrease the environmental and human health impacts of farming pesticide practices for crop protection. Integrated Crop Management (ICM) contains guidelines for farmers to use in enforcing actions for the production of safe agricultural products while also being environmentally conscious [303,[322], [323], [324]]. Furthermore, ICM comprises measures for the application of good agricultural practices (GAP), worker hygiene and safety, product security, full traceability of measurements, and specific environmental preservation actions [325]. ICM boosts the practice of complementary pest management approaches, including crop resistance to fungi and insects, biological control, and safety, while minimizing pesticide impacts on other agro-ecosystem components [326,327]. ICM permits pesticide usage only through an Integrated Pest Management (IPM) program in which certain criteria are used to select pesticides, specific instructions are followed for applying them to crops, and residue analysis is used as one of the enforcement tools [324,325]. Pesticides chosen for IPM are: (I) biologically effective (high selectivity, low risk of resistance, rapid impact, optimal residual effect, and plant tolerance); (II) user-friendly (lesser toxicity, optimum formulation, easy to use, safe packaging, extensive store stability); (III) environmentally compatible (rapid degradation in the atmosphere, low movement in the soil); and (IV) economically profitable [328]. It is clear that implementing an IPM system would help to decrease the effect of pesticides on the environment and human health while not affecting crop productivity or increasing the likelihood of crop losses [303]. Furthermore, it is important to promote collaboration and communication among stakeholders such as farmers, pesticide applicators, workers, researchers, and regulatory organizations. Such efforts can aid in the development of safer techniques and new solutions. The selection of less dangerous insecticides with reduced toxicity and environmental impact should be emphasized. Whenever possible, encourage the use of non-chemical alternatives such as biological controls, physical barriers, or cultural norms [329,330].

Storage, transportation, and pesticide application should all be done with extreme caution. Insecticides are used sparingly and judiciously, and integrated pest management is used to control pests [331,332]. In terms of farmer behavior, the health belief model (HBD) may be the best option [333]. Such issues can be resolved by implementing new policies and monitoring strategies for pesticide safety, as well as responding to external and internal stimuli [334,335]. External stimuli such as providing the required knowledge, guidance, and proper training can assist farmers in understanding pesticide use safely. Internal stimuli such as itching and headaches can influence a farmer's pesticide choice. Furthermore, a safety culture, such as the provision of proper clothing or personal protection equipment, can boost farmers' confidence and satisfaction [335]. The storage of pesticides is important for crop production, but they can be dangerous if used incorrectly. Safety issues Compliance is the substantial step from the protection of pesticides to storage, where specific precautionary measures or procedures are recommended [336,337]. Pesticide storage should be kept away from populated and sensitive areas such as water bodies and residential areas. Children and unauthorized individuals must not have access to the pesticides store. Pesticide storage should be kept away from populated and sensitive areas, such as water bodies and residential areas [338].

In addition, there have been significant changes in chemical crop protection in recent years, not just in terms of the development of new active ingredients but also in terms of the assessment of these chemicals environmental behavior, residues in crop plants, and potential toxicity to humans and the environment [[339], [340], [341]]. This is due to significant scientific progress in many disciplines, such as chemistry and molecular biology, which has greatly improved the process of finding new agrochemicals and re-evaluating the safety of pesticides currently in use. As a result, novel agrochemicals with improved safety profiles and novel modes of action with fewer side effects are the need of the hour.

**Fig. 5:**
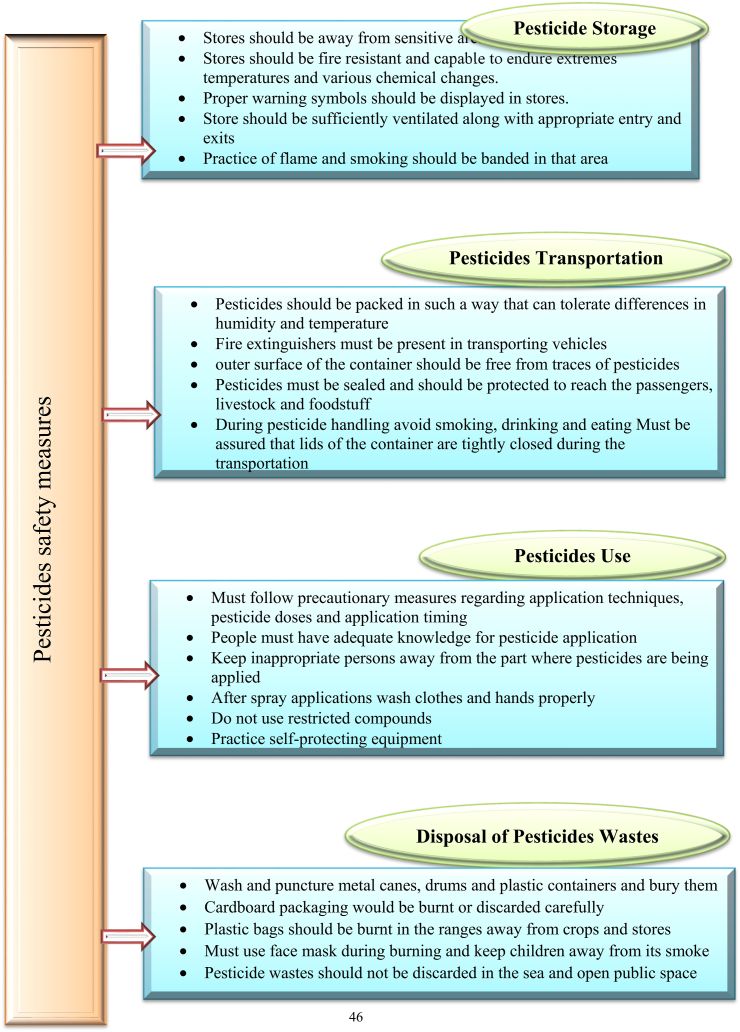
Different safety measures to control the exposure of pesticides to humans and the environment.

The possible harm that employees may endure as a result of interaction with pesticides while doing their tasks is referred to as pesticide exposure in occupational contexts. According to Damalas and Koutroubas (2016), this exposure can happen in a variety of industries, including manufacturing, pest control, horticulture, landscaping, and agriculture [213]. By mixing, applying, or handling these chemicals, workers in these sectors may come into direct contact with pesticides. Ingestion of tainted food or drink, absorption of pesticide dust or fumes, or other indirect exposure methods are also possible. Pesticide exposure can affect a worker's health in both the short and long term. Skin irritation, eye irritation, respiratory issues, nausea, vomiting, vertigo, and headaches are just a few of the acute side effects that might occur. In extreme circumstances, pesticide exposure can cause poisoning, which may have more serious consequences and lead to death [342,343]. In many nations, occupational safety laws and regulations have been implemented to safeguard workers from pesticide exposure. According to these rules, businesses are frequently required to provide suitable personal protective equipment (PPE), instruction on the proper handling and application of pesticides, and ongoing worker health monitoring [344,345]. IPM also emphasizes preventive strategies to efficiently manage pests while reducing dependency on chemical pesticides, such as enhancing sanitation and using biological controls. It's critical to regularly monitor and keep track of workers' pesticide exposure levels in order to spot any concerns and put the right controls in place. By conducting routine health checks, providing information, and monitoring compliance with safety rules, occupational health experts play a critical role in identifying and controlling the dangers of pesticide exposure at work. To preserve the health and wellbeing of employees in fields where these chemicals are employed, it is crucial to limit their exposure to pesticides in occupational contexts [345,346]. Fig. 5 has been depicted to explore various safety measures.

### 3.6 Legislation on pesticides

Handford et al. (2015) examined global pesticide legislation and discovered significant differences between countries and regions. In general, legislation in industrially developed countries is stricter, whereas developing countries lack the means and expertise for the application of legislation. The EU is specifically known for having some of the sternest pesticide laws in the world [347]. Three-quarters of all active ingredients formerly approved for use in the EU were banned during a major reevaluation of all active ingredients permitted for use in the EU between 1998 and 2009. Most nations practice maximum residue limits (MRLs) to regulate pesticides, which are defined as “the highest level of a pesticide residue that is legally tolerated in or on food or feed when pesticides are applied correctly”. MRLs differ dramatically between countries in some cases [347]. MRLs in the EU, Canada, the US, China, India, Japan, South Africa, and Australia were compared, and it was reported that MRLs in the EU were the lowest (representing the strictest legislation) and the highest reported in the US. Contrary to the EU, developing nations lack satisfactory legislation to efficiently regulate the usage, storage, labeling, disposal, and transportation of pesticides, owing to a lack of awareness of the risks involved as well as a lack of resources and knowledge to improve legislation. Different countries have different pesticide regulatory agencies; for example, in Brazil, three agencies share responsibility for pesticide legislation: agriculture, which assesses and records products; health, which evaluates human health threats; and environment, which evaluates environmental threats. Japan is one of the world's largest pesticide users. In Japan, MRLs are approved by the ministry of health, labor, and welfare [347,348]. The Institute for the Control of Agrochemicals, Ministry of Agriculture, was established under the Ministry of Agriculture (MOA) to regulate pesticide legislation in China. The USEPA (United States Environmental Protection Agency) is in charge of pesticide registration, which entails manufacturers submitting a comprehensive application for new substances and uses for pesticides that are already registered. However, researchers and journalists have questioned whether current legislation is effective in providing satisfactory protection for human health as well as the environment [349,350]. Meanwhile, Brazil is the world's largest pesticide consumer [351,352].

### 3.7 Future prospects

The prospect of pesticides comprises numerous potential risks, including health hazards, environmental contamination, and pesticide-resistant to pests [7,334]. There are a number of steps that may be taken to ensure the safety of both people and the environment, such as investing in the development of safer substitutes for chemical pesticides by governments, research organizations, and private businesses. In order to use less pesticides, IPM focuses on combining several pest control methods [353]. This includes keeping an eye out for pests, identifying them, establishing action thresholds, and putting pests under physical or mechanical control. Pesticide usage and sales should be subject to tight regulations enforced by regulatory oversight. Prior to sanctioning the use of any pesticide for commercial purposes, this requires carrying out extensive testing and risk analyses. To make sure that safety standards are being followed, regular monitoring and evaluation should also be done [354]. Additionally, education and training should be provided, especially to agricultural workers, farmers, and pesticide applicators, on the proper handling, storing, and use of pesticides. This entails knowing potential dangers and safety precautions, employing protective equipment, and adhering to label directions. To address the worldwide threats posed by pesticides, international coordination and cooperation between nations are essential [11].

## 4 Conclusion

An extensive analysis of pesticides revealed their effects on the environment, helped to identify possible health concerns, informed laws and policies, and helped to create sustainable alternatives to pesticide use. Pesticides can be ingested, inhaled, or absorbed via the skin. Assessing the cumulative impacts of numerous exposure routes and determining their proportional contributions to overall exposure can be difficult, leading to uncertainty in understanding the full impact. Pesticides have adverse impacts on the environment and human health. Educating stakeholders about these effects can help them make more informed decisions, lower risks, enhance safety protocols, practice environmental stewardship, protect the health of the community, and collaborate and communicate with each other. The scientific community can improve its understanding of pesticide impacts, encourage responsible usage, and help to design effective, science-based policies and practices that safeguard human health and the environment. Moreover, international pesticide monitoring policies must regularly adapt to the development of scientific information for human health protection and maintain a pollution-free environment. Different segments, for instance, government agencies, manufacturers, and NGOs, should come together and make protocols and policies regarding the development of pesticides with fewer side effects. The future of pesticides is expected to be driven by technological advancements, regulatory changes, consumer preferences, and increased awareness of the importance of sustainable agriculture. In conclusion, it is essential to balance the need for pest management against the potential hazards associated with pesticides.

## Ethics declaration

Review and/or approval by an ethics committee was not needed for this study because this literature review only used existing data from published studies and did not involve any direct experimentation or studies on living beings.

## Data availability statement

Data will be made available on request.

## CRediT authorship contribution statement

**Md Faruque Ahmad:** Writing – original draft, Formal analysis, Conceptualization. **Fakhruddin Ali Ahmad:** Supervision, Data curation. **Abdulrahman A. Alsayegh:** Resources, Formal analysis. **Md. Zeyaullah:** Funding acquisition, Data curation. **Abdullah M. AlShahrani:** Funding acquisition, Formal analysis. **Khursheed Muzammil:** Methodology. **Abdullah Ali Saati:** Resources. **Shadma Wahab:** Methodology, Data curation. **Ehab Y. Elbendary:** Resources, Formal analysis. **Nahla Kambal:** Visualization, Methodology. **Mohamed H. Abdelrahman:** Visualization, Resources. **Sohail Hussain:** Resources, Investigation.

## Declaration of competing interest

The authors declare that they have no known competing financial interests or personal relationships that could have appeared to influence the work reported in this paper.

## References

[1] B.A. Khan, M.A. Nadeem, H. Nawaz, M.M. Amin, G.H. Abbasi, M. Nadeem, M. Ali, M. Ameen, M.M. Javaid, R. Maqbool, Pesticides: impacts on agriculture productivity, environment, and management strategies, Emerging Contaminants and Plants: interactions, Adaptations and Remediation Technologies, Springer2023, pp. 109-134.

[2] Ahamad A., Kumar J. (2023). Pyrethroid pesticides: an overview on classification, toxicological assessment and monitoring. Journal of Hazardous Materials Advances.

[3] Elbialy Z.I., Assar D.H., Abdelnaby A., Asa S.A., Abdelhiee E.Y., Ibrahim S.S., Abdel-Daim M.M., Almeer R., Atiba A. (2021). Healing potential of Spirulina platensis for skin wounds by modulating bFGF, VEGF, TGF-ß1 and α-SMA genes expression targeting angiogenesis and scar tissue formation in the rat model. Biomed. Pharmacother..

[4] Trellu C., Vargas H.O., Mousset E., Oturan N., Oturan M.A. (2021). Electrochemical technologies for the treatment of pesticides. Curr. Opin. Electrochem..

[5] Singh R., Kumar N., Mehra R., Kumar H., Singh V.P. (2020). Progress and challenges in the detection of residual pesticides using nanotechnology based colorimetric techniques. Trends in Environmental Analytical Chemistry.

[6] Umapathi R., Ghoreishian S.M., Sonwal S., Rani G.M., Huh Y.S. (2022). Portable electrochemical sensing methodologies for on-site detection of pesticide residues in fruits and vegetables. Coord. Chem. Rev..

[7] Cech R., Zaller J.G., Lyssimachou A., Clausing P., Hertoge K., Linhart C. (2023). Pesticide drift mitigation measures appear to reduce contamination of non-agricultural areas, but hazards to humans and the environment remain. Sci. Total Environ..

[8] Fang L., Liao X., Jia B., Shi L., Kang L., Zhou L., Kong W. (2020). Recent progress in immunosensors for pesticides. Biosens. Bioelectron..

[9] Boedeker W., Watts M., Clausing P., Marquez E. (2020). The global distribution of acute unintentional pesticide poisoning: estimations based on a systematic review. BMC Publ. Health.

[10] Kaur R., Mavi G.K., Raghav S., Khan I. (2019). Pesticides classification and its impact on environment. Int. J. Curr. Microbiol. Appl. Sci.

[11] Rani L., Thapa K., Kanojia N., Sharma N., Singh S., Grewal A.S., Srivastav A.L., Kaushal J. (2021). An extensive review on the consequences of chemical pesticides on human health and environment. J. Clean. Prod..

[12] N.S. Singh, R. Sharma, T. Parween, P. Patanjali, Pesticide contamination and human health risk factor, Modern age environmental problems and their remediation, Springer2018, pp. 49-68.

[13] Frisoni G.B., Altomare D., Thal D.R., Ribaldi F., van der Kant R., Ossenkoppele R., Blennow K., Cummings J., van Duijn C., Nilsson P.M. (2022). The probabilistic model of Alzheimer disease: the amyloid hypothesis revised. Nat. Rev. Neurosci..

[14] Perrin L., Spinosi J., Chaperon L., Kab S., Moisan F., Ebaz A. (2021). Pesticides expenditures by farming type and incidence of Parkinson disease in farmers: a French nationwide study. Environ. Res..

[15] Sanborn M., Kerr K.J., Sanin L.H., Cole D.C., Bassil K.L., Vakil C. (2007). Non-cancer health effects of pesticides: systematic review and implications for family doctors. Can. Fam. Physician.

[16] Wang C., Yao X., Li X., Wang Q., Jiang N., Hu X., Lv H., Mu B., Wang J. (2024). Fosthiazate, a soil-applied nematicide, induces oxidative stress, neurotoxicity and transcriptome aberrations in earthworm (Eisenia fetida). J. Hazard Mater..

[17] Bhardwaj J.K., Mittal M., Saraf P., Kumari P. (2018).

[18] Foucault A., Vallet N., Ravalet N., Picou F., Bene M.C., Gyan E., Herault O. (2021). Occupational pesticide exposure increases risk of acute myeloid leukemia: a meta-analysis of case–control studies including 3,955 cases and 9,948 controls. Sci. Rep..

[19] Rafeeinia A., Asadikaram G., Moazed V., Darabi M.K. (2022). Organochlorine pesticides may induce leukemia by methylation of CDKN2B and MGMT promoters and histone modifications. Gene.

[20] Hernández-Mariano J.Á., Baltazar-Reyes M.C., Salazar-Martínez E., Cupul-Uicab L.A. (2022). Exposure to the pesticide DDT and risk of diabetes and hypertension: systematic review and meta-analysis of prospective studies. Int. J. Hyg Environ. Health.

[21] Popp J., Pető K., Nagy J. (2013). Pesticide productivity and food security. A review. Agron. Sustain. Dev..

[22] Tudi M., Daniel Ruan H., Wang L., Lyu J., Sadler R., Connell D., Chu C., Phung D.T. (2021). Agriculture development, pesticide application and its impact on the environment. Int. J. Environ. Res. Publ. Health.

[23] Kitchen L.W., Lawrence K.L., Coleman R.E. (2009). The role of the United States military in the development of vector control products, including insect repellents, insecticides, and bed nets. J. Vector Ecol..

[24] Hazra D.K., Karmakar R., Poi R., Bhattacharya S., Mondal S. (2017). Recent advances in pesticide formulations for eco-friendly and sustainable vegetable pest management: a review. Archives of Agriculture and Environmental Science.

[25] Zuma M., Arthur G., Coopoosamy R., Naidoo K. (2023). Incorporating cropping systems with eco-friendly strategies and solutions to mitigate the effects of climate change on crop production. Journal of Agriculture and Food Research.

[26] Yang Y., Guo Y., Jia X., Zhang Q., Mao J., Feng Y., Yin D., Zhao W., Zhang Y., Ouyang G. (2023). An ultrastable 2D covalent organic framework coating for headspace solid-phase microextraction of organochlorine pesticides in environmental water. J. Hazard Mater..

[27] Jintana S., Sming K., Krongtong Y., Thanyachai S. (2009). Cholinesterase activity, pesticide exposure and health impact in a population exposed to organophosphates. Int. Arch. Occup. Environ. Health.

[28] Yan H., Chen Y., Wang H., Jiao L., Chen H., Zhu C. (2023). Bismuth atom-doped gold aerogels for the detection of acetylcholinesterase activity and organophosphorus inhibitor. Chem. Eng. J..

[29] Yadav I.C., Devi N.L. (2017). Pesticides classification and its impact on human and environment. Environ. Sci. Eng..

[30] Yan D., Zhang Y., Liu L., Yan H. (2016). Pesticide exposure and risk of Alzheimer's disease: a systematic review and meta-analysis. Sci. Rep..

[31] Wahab S., Muzammil K., Nasir N., Khan M.S., Ahmad M.F., Khalid M., Ahmad W., Dawria A., Reddy L.K.V., Busayli A.M. (2022). Advancement and new trends in analysis of pesticide residues in food: a comprehensive review. Plants.

[32] Fahimi-Kashani N., Hormozi-Nezhad M.R. (2016). Gold-nanoparticle-based colorimetric sensor array for discrimination of organophosphate pesticides. Anal. Chem..

[33] Mwila K., Burton M., Van Dyk J., Pletschke B. (2013). The effect of mixtures of organophosphate and carbamate pesticides on acetylcholinesterase and application of chemometrics to identify pesticides in mixtures. Environ. Monit. Assess..

[34] Sepahi S., Gerayli S., Delirrad M., Taghavizadeh Yazdi M.E., Zare‐Zardini H., Bushehri B., Ghorani‐Azam A. (2023). Biochemical responses as early and reliable biomarkers of organophosphate and carbamate pesticides intoxication: a systematic literature review. J. Biochem. Mol. Toxicol..

[35] Drum C. (1980).

[36] Zhang X., Huang Y., Chen W.-J., Wu S., Lei Q., Zhou Z., Zhang W., Mishra S., Bhatt P., Chen S. (2023). Environmental occurrence, toxicity concerns, and biodegradation of neonicotinoid insecticides. Environ. Res..

[37] Zaharia R., Trotuș E., Trașcă G., Georgescu E., Șapcaliu A., Fătu V., Petrișor C., Mincea C. (2023). Impact of seed treatment with imidacloprid, clothianidin and thiamethoxam on soil, plants, bees and hive products. Agriculture.

[38] Costas-Ferreira C., Durán R., Faro L.R. (2022). Toxic effects of glyphosate on the nervous system: a systematic review. Int. J. Mol. Sci..

[39] Saunders L.E., Pezeshki R. (2015). Glyphosate in runoff waters and in the root-zone: a review. Toxics.

[40] Leal J.F.L., Borella J., dos Santos Souza A., Langaro A.C., de Moura Carneiro R., de Souza da Silva G., de Oliveira Junior F.F., de Souza F.R., Machado A.F.L., de Pinho C.F. (2023). Photosystem II-and photosystem I-inhibitor herbicides-driven changes in the dynamics of photosynthetic energy dissipation of Conyza spp. Acta Physiol. Plant..

[41] Shahinasi E., Brahushi F., Devolli A., Kodra M. (2017). The ecotoxicology of pesticides group of triazole and their use to control apple scab (Venturia inaequalis). Journal of Hygienic Engineering and Design.

[42] Cloyd R.A., Bethke J.A., Cowles R.S. (2011). Systemic insecticides and their use in ornamental plant systems. Floric. Ornam. Biotechnol..

[43] Alengebawy A., Abdelkhalek S.T., Qureshi S.R., Wang M.-Q. (2021). Heavy metals and pesticides toxicity in agricultural soil and plants: ecological risks and human health implications. Toxics.

[44] Chagnon M., Kreutzweiser D., Mitchell E.A., Morrissey C.A., Noome D.A., Van der Sluijs J.P. (2015). Risks of large-scale use of systemic insecticides to ecosystem functioning and services. Environ. Sci. Pollut. Res..

[45] Sulaiman N.S., Rovina K., Joseph V.M. (2019). Classification, extraction and current analytical approaches for detection of pesticides in various food products. Journal of Consumer Protection and Food Safety.

[46] Simon-Delso N., Amaral-Rogers V., Belzunces L.P., Bonmatin J.-M., Chagnon M., Downs C., Furlan L., Gibbons D.W., Giorio C., Girolami V. (2015). Systemic insecticides (neonicotinoids and fipronil): trends, uses, mode of action and metabolites. Environ. Sci. Pollut. Control Ser..

[47] Wu T., Zhao K., Liu S., Bao Z., Zhang C., Wu Y., Song R., Gu Y., Gao Y., Du F. (2023). Promising nanocarriers endowing non-systemic pesticides with upward translocation ability and microbial community enrichment effects in soil. Chem. Eng. J..

[48] Gerbig S., Brunn H.E., Spengler B., Schulz S. (2015). Spatially resolved investigation of systemic and contact pesticides in plant material by desorption electrospray ionization mass spectrometry imaging (DESI-MSI). Anal. Bioanal. Chem..

[49] Satpathy S., Gotyal B., Babu V.R. (2020). Role of novel insecticides in crop protection and their selectivity to natural enemies: a review. J. Environ. Biol..

[50] Łozowicka B., Kaczyński P., Mojsak P., Rusiłowska J., Beknazarova Z., Ilyasova G., Absatarova D. (2020). Systemic and non-systemic pesticides in apples from Kazakhstan and their impact on human health. J. Food Compos. Anal..

[51] Yadav I.C., Devi N.L. (2017). Pesticides classification and its impact on human and environment. Environ. Sci. Eng..

[52] M.A. Dar, G. Kaushik, Classification of pesticides and loss of crops due to creepy crawlers, Pesticides in the Natural Environment, Elsevier2022, pp. 1-21.

[53] Y. Abubakar, H. Tijjani, C. Egbuna, C.O. Adetunji, S. Kala, T.L. Kryeziu, J.C. Ifemeje, K.C. Patrick-Iwuanyanwu, Pesticides, History, and Classification, Natural Remedies for Pest, Disease and Weed Control, Elsevier2020, pp. 29-42.

[54] I. Alizadeh, M.A. Gorouhi, A.A. Afshar, M. Faraji, M.N. Pour, F.P. Risk, Please Cite This Article as.

[55] Leoci R., Ruberti M. (2021). Pesticides: an overview of the current health problems of their use. J. Geosci. Environ. Protect..

[56] Tubiello F., Wanner N., Asprooth L., Mueller M., Ignaciuk A., Khan A., Rosero Moncayo J. (2021).

[57] Kim K.-H., Kabir E., Jahan S.A. (2017). Exposure to pesticides and the associated human health effects. Sci. Total Environ..

[58] Fishel F.M., Ferrell J.A. (2010). Managing pesticide drift. Environ. Data Inf. Serv..

[59] M. Butu, S. Rodino, A. Butu, Biopesticide formulations-current challenges and future perspectives, Biopesticides, Elsevier2022, pp. 19-29.

[60] Nagy K., Duca R.C., Lovas S., Creta M., Scheepers P.T., Godderis L., Ádám B. (2020). Systematic review of comparative studies assessing the toxicity of pesticide active ingredients and their product formulations. Environ. Res..

[61] Pose-Juan E., Rial-Otero R., Martínez-Carballo E., López-Periago E., Simal-Gándara J. (2009). Determination of metalaxyl and identification of adjuvants in wettable powder pesticide technical formulas. Anal. Bioanal. Chem..

[62] Hazra D.K., Purkait A. (2019). Role of pesticide formulations for sustainable crop protection and environment management: a review. J. Pharmacogn. Phytochem..

[63] Stafford T.R., Best L.B., Fischer D.L. (1996). Effects of different formulations of granular pesticides on birds. Environ. Toxicol. Chem.: Int. J..

[64] Shapiro-Ilan D.I., Gouge D.H., Piggott S.J., Fife J.P. (2006). Application technology and environmental considerations for use of entomopathogenic nematodes in biological control. Biol. Control.

[65] Singh A., Dhiman N., Kar A.K., Singh D., Purohit M.P., Ghosh D., Patnaik S. (2020). Advances in controlled release pesticide formulations: prospects to safer integrated pest management and sustainable agriculture. J. Hazard Mater..

[66] Alonso M.L., Laza J.M., Alonso R.M., Jiménez R.M., Vilas J.L., Fañanás R. (2014). Pesticides microencapsulation. A safe and sustainable industrial process. J. Chem. Technol. Biotechnol..

[67] Zhao X., Cui H., Wang Y., Sun C., Cui B., Zeng Z. (2017). Development strategies and prospects of nano-based smart pesticide formulation. J. Agric. Food Chem..

[68] Mesnage R., Antoniou M.N. (2018). Ignoring adjuvant toxicity falsifies the safety profile of commercial pesticides. Front. Public Health.

[69] Alavanja M.C. (2009). Introduction: pesticides use and exposure, extensive worldwide. Rev. Environ. Health.

[70] Pereira J.L., Antunes S.C., Castro B.B., Marques C.R., Gonçalves A.M., Gonçalves F., Pereira R. (2009). Toxicity evaluation of three pesticides on non-target aquatic and soil organisms: commercial formulation versus active ingredient. Ecotoxicology.

[71] Altaf R., Ullah Z., Darko D.A., Iqbal A., Khan M.S., Asif M. (2022). Molecularly imprinted polymers for the detection of chlorpyrifos (an organophosphate pesticide). ASEAN Journal of Science and Engineering.

[72] Wongta A., Sawang N., Tongjai P., Jatiket M., Hongsibsong S. (2022). The assessment of organophosphate pesticide exposure among school children in four regions of Thailand: analysis of dialkyl phosphate metabolites in students' urine and organophosphate pesticide residues in vegetables for school lunch. Toxics.

[73] Hites R.A. (2021). The rise and fall of chlorpyrifos in the United States. Environ. Sci. Technol..

[74] Nandi N.K., Vyas A., Akhtar M.J., Kumar B. (2022). The growing concern of chlorpyrifos exposures on human and environmental health. Pestic. Biochem. Physiol..

[75] Giddings J.M., Williams W.M., Solomon K.R., Giesy J.P. (2014). Ecological Risk Assessment for Chlorpyrifos in Terrestrial and Aquatic Systems in the United States.

[76] Mamy L., Barriuso E., Gabrielle B. (2005). Environmental fate of herbicides trifluralin, metazachlor, metamitron and sulcotrione compared with that of glyphosate, a substitute broad spectrum herbicide for different glyphosate‐resistant crops. Pest Manag. Sci.: formerly Pesticide Science.

[77] Gill J.P.K., Sethi N., Mohan A., Datta S., Girdhar M. (2018). Glyphosate toxicity for animals. Environ. Chem. Lett..

[78] Klátyik S., Simon G., Oláh M., Mesnage R., Antoniou M.N., Zaller J.G., Székács A. (2023). Terrestrial ecotoxicity of glyphosate, its formulations, and co-formulants: evidence from 2010–2023. Environ. Sci. Eur..

[79] Zhou Q., Kan B., Jian X., Zhang W., Liu H., Zhang Z. (2013). Paraquat poisoning by skin absorption: two case reports and a literature review. Exp. Ther. Med..

[80] Wu M.-R., Hsiao C.-Y., Cheng C.-H., Liao F.-C., Chao C.-L., Chen C.-Y., Yeh H.-I., Su M.-I. (2018). Is endotracheal intubation a non-beneficial treatment in patients with respiratory failure due to paraquat poisoning?. PLoS One.

[81] Subbiah R., Tiwari R.R. (2021). The herbicide paraquat-induced molecular mechanisms in the development of acute lung injury and lung fibrosis. Crit. Rev. Toxicol..

[82] Kim D., Burkett-Cadena N.D., Reeves L.E. (2022). Pollinator biological traits and ecological interactions mediate the impacts of mosquito-targeting malathion application. Sci. Rep..

[83] Elmorsy E., Al-Ghafari A., Al Doghaither H., Salama M., Carter W.G. (2022). An investigation of the neurotoxic effects of malathion, chlorpyrifos, and paraquat to different brain regions. Brain Sci..

[84] Uikey S. (2015). Effect of malathion toxicity on fresh water fish Labeo rohita. International Journal of Applied and Universal Research.

[85] Lotti M., Moretto A. (2005). Organophosphate-induced delayed polyneuropathy. Toxicol. Rev..

[86] Kalyabina V.P., Esimbekova E.N., Kopylova K.V., Kratasyuk V.A. (2021). Pesticides: formulants, distribution pathways and effects on human health–a review. Toxicol Rep.

[87] Oliver R.P., Beckerman J.L. (2022).

[88] Karaca M., Fischer B.C., Willenbockel C.T., Tralau T., Marx-Stoelting P., Bloch D. (2021). Effects of co-formulants on the absorption and secretion of active substances in plant protection products in vitro. Arch. Toxicol..

[89] M.K. Mishra, S.K. Mishra, L. Pandey, A. Kumar, Pesticides and Their Formulations.

[90] Gradiski D., Vinot J., Zissu D., Limasset J., Lafontaine M. (1983). The carcinogenic effect of a series of petroleum-derived oils on the skin of mice. Environ. Res..

[91] Chokwe T., Okonkwo J., Sibali L. (2017). Distribution, exposure pathways, sources and toxicity of nonylphenol and nonylphenol ethoxylates in the environment. WaterSA.

[92] Uğuz C., İşcan M., Togan İ. (2009). Alkylphenols in the environment and their adverse effects on living organisms. Kocatepe Veterinary Journal.

[93] Guenther K., Heinke V., Thiele B., Kleist E., Prast H., Raecker T. (2003). Response to comments on “Endocrine disrupting nonylphenols are ubiquitous in food”. Environ. Sci. Technol..

[94] Mesnage R., Benbrook C., Antoniou M.N. (2019). Insight into the confusion over surfactant co-formulants in glyphosate-based herbicides. Food Chem. Toxicol..

[95] de la Cruz Quiroz R., Cruz Maldonado J.J., Rostro Alanis M.d.J., Torres J.A., Parra Saldívar R. (2019). Fungi-based biopesticides: shelf-life preservation technologies used in commercial products. J. Pest. Sci..

[96] Tominack R.L., Tominack R. (2000). Herbicide formulations. J. Toxicol. Clin. Toxicol..

[97] Kwak K., Paek D., Park J.T. (2020). Occupational exposure to formaldehyde and risk of lung cancer: a systematic review and meta‐analysis. Am. J. Ind. Med..

[98] J.S. Leal, M. Garcia, I. Ribosa, F. Comelles, Environmental risk assessment of ethoxylated non-ionic surfactants, Surfactants in Solution, CRC Press2020, pp. 379-391.

[99] Cetin H., Demir E., Kocaoglu S., Kaya B. (2010). Insecticidal activity of some synthetic pyrethroids with different rates of piperonyl butoxide (PBO) combinations on Drosophila melanogaster (Diptera: drosophilidae). Ekoloji.

[100] Bubić Pajić N.Z., Todosijević M.N., Vuleta G.M., Cekić N.D., Dobričić V.D., Vučen S.R., Čalija B.R., Lukić M.Ž., Ilić T.M., Savić S.D. (2017). Alkyl polyglucoside vs. ethoxylated surfactant-based microemulsions as vehicles for two poorly water-soluble drugs: physicochemical characterization and in vivo skin performance. Acta Pharm..

[101] Geetha D., Tyagi R. (2012). Alkyl poly glucosides (APGs) surfactants and their properties: a review. Tenside Surfactants Deterg..

[102] Jablonkai I. (2011). Herbicides—Mechanisms and Mode of Action.

[103] Tarazona J.V., Tiramani M., Reich H., Pfeil R., Istace F., Crivellente F. (2017). Glyphosate toxicity and carcinogenicity: a review of the scientific basis of the European Union assessment and its differences with IARC. Arch. Toxicol..

[104] Pretty J., Hine R. (2002).

[105] Corniani N., Velini E.D., Silva F.M., Nanayakkara N.D., Witschel M., Dayan F.E. (2014). Novel bioassay for the discovery of inhibitors of the 2-C-Methyl-D-erythritol 4-phosphate (MEP) and terpenoid pathways leading to carotenoid biosynthesis. PLoS One.

[106] De Roos A.J., Blair A., Rusiecki J.A., Hoppin J.A., Svec M., Dosemeci M., Sandler D.P., Alavanja M.C. (2005). Cancer incidence among glyphosate-exposed pesticide applicators in the Agricultural Health Study. Environ. Health Perspect..

[107] Thrall P.H., Oakeshott J.G., Fitt G., Southerton S., Burdon J.J., Sheppard A., Russell R.J., Zalucki M., Heino M., Ford Denison R. (2011). Evolution in agriculture: the application of evolutionary approaches to the management of biotic interactions in agro‐ecosystems. Evolutionary applications.

[108] Beffa R., Menne H., Köcher H. (2019). Herbicide resistance action committee (HRAC): herbicide classification, resistance evolution, survey, and resistance mitigation activities. Modern crop protection compounds.

[109] Traxler C., Gaines T.A., Küpper A., Luemmen P., Dayan F.E. (2023). The nexus between reactive oxygen species and the mechanism of action of herbicides. J. Biol. Chem..

[110] He B., Hu Y., Wang W., Yan W., Ye Y. (2022). The progress towards novel herbicide modes of action and targeted herbicide development. Agronomy.

[111] Grossmann K., Niggeweg R., Christiansen N., Looser R., Ehrhardt T. (2010). The herbicide saflufenacil (Kixor™) is a new inhibitor of protoporphyrinogen IX oxidase activity. Weed Sci..

[112] Wang X., Luo M.-J., Wang Y.-X., Han W.-Q., Miu J.-X., Luo X.-P., Zhang A.-D., Kuang Y. (2022). Design, synthesis, and herbicidal activity of indole-3-carboxylic acid derivatives as potential transport inhibitor response 1 antagonists. Front. Chem..

[113] Governa P., Bernardini G., Braconi D., Manetti F., Santucci A., Petricci E. (2022). Survey on the recent advances in 4-hydroxyphenylpyruvate dioxygenase (HPPD) inhibition by diketone and triketone derivatives and congeneric compounds: structural analysis of HPPD/inhibitor complexes and structure–activity relationship considerations. J. Agric. Food Chem..

[114] Götz T., Böger P. (2004). The very-long-chain fatty acid synthase is inhibited by chloroacetamides. Z. Naturforsch. C Biosci..

[115] Zohar Y., Einav M., Chipman D.M., Barak Z.e. (2003). Acetohydroxyacid synthase from Mycobacterium avium and its inhibition by sulfonylureas and imidazolinones. Biochim. Biophys. Acta Protein Proteonomics.

[116] Rajashekar Y., Tonsing N., Shantibala T., Manjunath J.R. (2016). 2, 3-Dimethylmaleic anhydride (3, 4-Dimethyl-2, 5-furandione): a plant derived insecticidal molecule from Colocasia esculenta var. esculenta (L.) Schott. Sci. Rep..

[117] Nauen R. (2006). Insecticide mode of action: return of the ryanodine receptor. Pest Manag. Sci.: formerly Pesticide Science.

[118] Elbert A., Nauen R., Salmon E. (2008). Resistance management guidelines for the new ketoenol insecticide Movento. Bayer CropScience Journal.

[119] Songa E.A., Okonkwo J.O. (2016). Recent approaches to improving selectivity and sensitivity of enzyme-based biosensors for organophosphorus pesticides: a review. Talanta.

[120] Sparks T.C., Nauen R. (2015). IRAC: mode of action classification and insecticide resistance management. Pestic. Biochem. Physiol..

[121] Jayaraj R., Megha P., Sreedev P. (2016). Organochlorine pesticides, their toxic effects on living organisms and their fate in the environment. Interdiscipl. Toxicol..

[122] Wipff J.K. (2002). Proceedings of Scientific Methods Workshop: Ecological and Agronomic Consequences of Gene Flow from Transgenic Crops to Wild Relatives.

[123] Tanaka K. (2019). Studies on the metabolism, mode of action, and development of insecticides acting on the GABA receptor. J. Pestic. Sci..

[124] Sparks T.C., Storer N., Porter A., Slater R., Nauen R. (2021). Insecticide resistance management and industry: the origins and evolution of the Insecticide Resistance Action Committee (IRAC) and the mode of action classification scheme. Pest Manag. Sci..

[125] Fukuto T.R. (1990). Mechanism of action of organophosphorus and carbamate insecticides. Environ. Health Perspect..

[126] Rezende-Teixeira P., Dusi R.G., Jimenez P.C., Espindola L.S., Costa-Lotufo L.V. (2022). What can we learn from commercial insecticides? Efficacy, toxicity, environmental impacts, and future developments. Environ. Pollut..

[127] Demaeght P., Osborne E.J., Odman-Naresh J., Grbić M., Nauen R., Merzendorfer H., Clark R.M., Van Leeuwen T. (2014). High resolution genetic mapping uncovers chitin synthase-1 as the target-site of the structurally diverse mite growth inhibitors clofentezine, hexythiazox and etoxazole in Tetranychus urticae. Insect Biochem. Mol. Biol..

[128] Ruder F., Kayser H. (1994). The thiourea insecticide diafenthiuron inhibits mitochondrial ATPase in vitro and in vivo by its carbodiimide product. Biochem. Soc. Trans..

[129] Pérez M.E., Soto E., Vega R. (1991). Streptomycin blocks the postsynaptic effects of excitatory amino acids on the vestibular system primary afferents. Brain Res..

[130] Carr J.F., Gregory S.T., Dahlberg A.E. (2005). Severity of the streptomycin resistance and streptomycin dependence phenotypes of ribosomal protein S12 of Thermus thermophilus depends on the identity of highly conserved amino acid residues. J. Bacteriol..

[131] Sioud M., Boudabous A., Cekaite L. (2009). Transcriptional responses of Bacillus subtillis and thuringiensis to antibiotics and anti-tumour drugs. Int. J. Mol. Med..

[132] White P.M., Potter T.L., Culbreath A.K. (2010). Fungicide dissipation and impact on metolachlor aerobic soil degradation and soil microbial dynamics. Sci. Total Environ..

[133] Aponte C., Marañón T., García L.V. (2010). Microbial C, N and P in soils of Mediterranean oak forests: influence of season, canopy cover and soil depth. Biogeochemistry.

[134] Oruc H.H., Carisse O. (2010). Fungicides and Their Effects on Animals.

[135] Burns E.R., Buchanan G.A., Carter M.C. (1971). Inhibition of carotenoid synthesis as a mechanism of action of amitrole, dichlormate, and pyriclor. Plant Physiol..

[136] Sui G., Zhang W., Zhou K., Li Y., Zhang B., Xu D., Zou Y., Zhou W. (2017). Trialkylamine derivatives containing a triazole moiety as promising ergosterol biosynthesis inhibitor: design, synthesis, and antifungal activity. Chem. Pharm. Bull..

[137] Zhou Y., Xu J., Zhu Y., Duan Y., Zhou M. (2016). Mechanism of action of the benzimidazole fungicide on Fusarium graminearum: interfering with polymerization of monomeric tubulin but not polymerized microtubule. Phytopathology.

[138] Wang F., Li X., Zhu L., Du Z., Zhang C., Wang J., Wang J., Lv D. (2018). Responses of soil microorganisms and enzymatic activities to azoxystrobin in cambisol. Pol. J. Environ. Stud..

[139] Silva L.M., De Souza D. (2017). Ziram herbicide determination using a polished silver solid amalgam electrode. Electrochim. Acta.

[140] Luo B., Ning Y. (2022). Comprehensive overview of carboxamide derivatives as succinate dehydrogenase inhibitors. J. Agric. Food Chem..

[141] Bolognesi C., Merlo F. (2019).

[142] Rath N.C., Rasaputra K.S., Liyanage R., Huff G.R., Huff W.E. (2011). Pesticides in the Modern World—Effects of Pesticides Exposure 2011.

[143] Zheng R., García-González J., Romero-del Rey R., López-Villén A., García-Alvarez R., Fadul-Calderon R., Requena-Mullor M., Alarcón-Rodríguez R. (2023). Occupational exposure to pesticides as a risk factor for sleep disorders. Int. J. Environ. Res. Publ. Health.

[144] Dara D., Drabovich A.P. (2023). Assessment of risks, implications, and opportunities of waterborne neurotoxic pesticides. J. Environ. Sci..

[145] Liu H., Reynolds G.P., Wei X. (2023). The influence of agricultural work and plasma uric acid on hospital admission for alzheimer's disease. J. Alzheim. Dis..

[146] Li Y., Zhang C., Yin Y., Cui F., Cai J., Chen Z., Jin Y., Robson M.G., Li M., Ren Y. (2014). Neurological effects of pesticide use among farmers in China. Int. J. Environ. Res. Publ. Health.

[147] Hayden K.M., Norton M.C., Darcey D., Østbye T., Zandi P.P., Breitner J., Welsh-Bohmer K. (2010). Occupational exposure to pesticides increases the risk of incident AD: the Cache County study. Neurology.

[148] Health C.S.o. (1994). Aging, the Canadian study of health and aging* risk factors for Alzheimer's disease in Canada. Neurology.

[149] Richardson J.R., Roy A., Shalat S.L., Von Stein R.T., Hossain M.M., Buckley B., Gearing M., Levey A.I., German D.C. (2014). Elevated serum pesticide levels and risk for Alzheimer disease. JAMA Neurol..

[150] Hernández A.F., Gil F., Lacasaña M. (2017). Toxicological interactions of pesticide mixtures: an update. Arch. Toxicol..

[151] González-Alzaga B., Lacasaña M., Aguilar-Garduño C., Rodríguez-Barranco M., Ballester F., Rebagliato M., Hernández A. (2014). A systematic review of neurodevelopmental effects of prenatal and postnatal organophosphate pesticide exposure. Toxicol. Lett..

[152] Carles C., Bouvier G., Lebailly P., Baldi I. (2017). Use of job-exposure matrices to estimate occupational exposure to pesticides: a review. J. Expo. Sci. Environ. Epidemiol..

[153] Yang Y., Zhou S., Xing Y., Yang G., You M. (2023). Impact of pesticides exposure during neurodevelopmental period on autism spectrum disorders–A focus on gut microbiota. Ecotoxicol. Environ. Saf..

[154] A. AC02133685, Guidance Document for the Conduct of Studies of Occupational Exposure to Pesticides during Agricultural Application, OECD1997.

[155] Le Couteur D.G., McLean A.J., Taylor M.C., Woodham B.L., Board P. (1999). Pesticides and Parkinson's disease. Biomed. Pharmacother..

[156] Rajawat N.K., Bhardwaj K., Mathur N. (2023). Risk of Parkinson disease associated with pesticide exposure and protection by probiotics. Mater. Today: Proc..

[157] Binienda Z.K., Sarkar S., Mohammed-Saeed L., Gough B., Beaudoin M.A., Ali S.F., Paule M.G., Imam S.Z. (2013). Chronic exposure to rotenone, a dopaminergic toxin, results in peripheral neuropathy associated with dopaminergic damage. Neurosci. Lett..

[158] Pezzoli G., Cereda E. (2013). Exposure to pesticides or solvents and risk of Parkinson disease. Neurology.

[159] Priyadarshi A., Khuder S.A., Schaub E.A., Shrivastava S. (2000). A meta-analysis of Parkinson's disease and exposure to pesticides. Neurotoxicology.

[160] D. Colle, M. Farina, Oxidative stress in paraquat-induced damage to nervous tissues, Toxicology, Elsevier2021, pp. 69-78.

[161] Zhang X.-f., Thompson M., Xu Y.-h. (2016). Multifactorial theory applied to the neurotoxicity of paraquat and paraquat-induced mechanisms of developing Parkinson's disease. Lab. Invest..

[162] Martínez-Chacón G., Yakhine-Diop S.M., González-Polo R.A., Bravo-San Pedro J.M., Pizarro-Estrella E., Niso-Santano M., Fuentes J.M. (2021).

[163] Alizadeh S., Anani-Sarab G., Amiri H., Hashemi M. (2022). Paraquat induced oxidative stress, DNA damage, and cytotoxicity in lymphocytes. Heliyon.

[164] See W.Z.C., Naidu R., Tang K.S. (2022). Cellular and molecular events leading to paraquat-induced apoptosis: mechanistic insights into Parkinson's disease pathophysiology. Mol. Neurobiol..

[165] Vaccari C., El Dib R., Gomaa H., Lopes L.C., de Camargo J.L. (2019). Paraquat and Parkinson's disease: a systematic review and meta-analysis of observational studies. J. Toxicol. Environ. Health, Part A B.

[166] Brouwer M., Koeman T., van den Brandt P.A., Kromhout H., Schouten L.J., Peters S., Huss A., Vermeulen R. (2015). Occupational exposures and Parkinson's disease mortality in a prospective Dutch cohort. Occup. Environ. Med..

[167] Ratner M.H., Farb D.H., Ozer J., Feldman R.G., Durso R. (2014). Younger age at onset of sporadic Parkinson's disease among subjects occupationally exposed to metals and pesticides. Interdiscipl. Toxicol..

[168] Kumar A., Margekar S.L., Margekar P., Margekar V. (2018). Recent advances in management of organophosphate & carbamate poisoning. Indian J. Med. Specialities.

[169] Colovic M.B., Krstic D.Z., Lazarevic-Pasti T.D., Bondzic A.M., Vasic V.M. (2013). Acetylcholinesterase inhibitors: pharmacology and toxicology. Curr. Neuropharmacol..

[170] Bagchi M., Ghosh S., Bagchi D., Hassoun E., Stohs S. (1995). Protective effects of lazaroid U74389F (16-desmethyl tirilazad) on endrin-induced lipid peroxidation and DNA damage in brain and liver and regional distribution of catalase activity in rat brain. Free Radic. Biol. Med..

[171] Li S.H., Graham B.M. (2017). Why are women so vulnerable to anxiety, trauma-related and stress-related disorders? The potential role of sex hormones. Lancet Psychiatr..

[172] Evangelista de Duffard A., Duffard R. (1996). Behavioral toxicology, risk assessment, and chlorinated hydrocarbons. Environ. Health Perspect..

[173] Seralini G., Jungers G. (2021). Endocrine disruptors also function as nervous disruptors and can be renamed endocrine and nervous disruptors (ENDs). Toxicol Rep.

[174] Gargouri B., Yousif N.M., Attaai A., Bouchard M., Chtourou Y., Fiebich B.L., Fetoui H. (2018). Pyrethroid bifenthrin induces oxidative stress, neuroinflammation, and neuronal damage, associated with cognitive and memory impairment in murine hippocampus. Neurochem. Int..

[175] Yang C., Lim W., Song G. (2020). Mediation of oxidative stress toxicity induced by pyrethroid pesticides in fish. Comp. Biochem. Physiol. C Toxicol. Pharmacol..

[176] Pearson J.N., Patel M. (2016). The role of oxidative stress in organophosphate and nerve agent toxicity. Ann. N. Y. Acad. Sci..

[177] Sasaki N., Jones L.E., Morse G.S., Carpenter D.O., Environment A.T.F.o.t. (2023). Mixture effects of polychlorinated biphenyls (PCBs) and three organochlorine pesticides on cognitive function in Mohawk adults at Akwesasne. Int. J. Environ. Res. Publ. Health.

[178] Lee D.-H., Lind P.M., Jacobs D.R., Salihovic S., van Bavel B., Lind L. (2016). Association between background exposure to organochlorine pesticides and the risk of cognitive impairment: a prospective study that accounts for weight change. Environ. Int..

[179] Baldi I., Gruber A., Rondeau V., Lebailly P., Brochard P., Fabrigoule C. (2011). Neurobehavioral effects of long-term exposure to pesticides: results from the 4-year follow-up of the PHYTONER study. Occup. Environ. Med..

[180] Bouchard M.F., Chevrier J., Harley K.G., Kogut K., Vedar M., Calderon N., Trujillo C., Johnson C., Bradman A., Barr D.B. (2011). Prenatal exposure to organophosphate pesticides and IQ in 7-year-old children. Environ. Health Perspect..

[181] Engel S.M., Wetmur J., Chen J., Zhu C., Barr D.B., Canfield R.L., Wolff M.S. (2011). Prenatal exposure to organophosphates, paraoxonase 1, and cognitive development in childhood. Environ. Health Perspect..

[182] Rauh V., Arunajadai S., Horton M., Perera F., Hoepner L., Barr D.B., Whyatt R. (2011). Seven-year neurodevelopmental scores and prenatal exposure to chlorpyrifos, a common agricultural pesticide. Environ. Health Perspect..

[183] Wahab S., Alshahrani M.Y., Ahmad M.F., Abbas H. (2021). Current trends and future perspectives of nanomedicine for the management of colon cancer. Eur. J. Pharmacol..

[184] Ahmad M.F. (2020). Ganoderma lucidum: a rational pharmacological approach to surmount cancer. J. Ethnopharmacol..

[185] de Graaf L., Boulanger M., Bureau M., Bouvier G., Meryet-Figuiere M., Tual S., Lebailly P., Baldi I. (2022). Occupational pesticide exposure, cancer and chronic neurological disorders: a systematic review of epidemiological studies in greenspace workers. Environ. Res..

[186] Cavalier H., Trasande L., Porta M. (2023). Exposures to pesticides and risk of cancer: evaluation of recent epidemiological evidence in humans and paths forward. Int. J. Cancer.

[187] Rebouillat P., Vidal R., Cravedi J.-P., Taupier-Letage B., Debrauwer L., Gamet-Payrastre L., Touvier M., Deschasaux-Tanguy M., Latino-Martel P., Hercberg S. (2021). Prospective association between dietary pesticide exposure profiles and postmenopausal breast-cancer risk in the NutriNet-Santé cohort. Int. J. Epidemiol..

[188] Sasikala S., Minu Jenifer M., Velavan K., Sakthivel M., Sivasamy R., Fenwick Antony E. (2023). Predicting the relationship between pesticide genotoxicity and breast cancer risk in South Indian women in in vitro and in vivo experiments. Sci. Rep..

[189] Ferro R., Parvathaneni A., Patel S., Cheriyath P. (2012). Pesticides and breast cancer. Adv. Breast Cancer Res..

[190] Ventura C., Venturino A., Miret N., Randi A., Rivera E., Núñez M., Cocca C. (2015). Chlorpyrifos inhibits cell proliferation through ERK1/2 phosphorylation in breast cancer cell lines. Chemosphere.

[191] Rivero J., Luzardo O.P., Henríquez-Hernández L.A., Machín R.P., Pestano J., Zumbado M., Boada L.D., Camacho M., Valerón P.F. (2015). In vitro evaluation of oestrogenic/androgenic activity of the serum organochlorine pesticide mixtures previously described in a breast cancer case–control study. Sci. Total Environ..

[192] Wolff M.S., Toniolo P.G., Lee E.W., Rivera M., Dubin N. (1993). Blood levels of organochlorine residues and risk of breast cancer. J. Natl. Cancer Inst.: J. Natl. Cancer Inst..

[193] Cohn B.A., Wolff M.S., Cirillo P.M., Sholtz R.I. (2007). DDT and breast cancer in young women: new data on the significance of age at exposure. Environ. Health Perspect..

[194] Arrebola J.P., Belhassen H., Artacho-Cordón F., Ghali R., Ghorbel H., Boussen H., Perez-Carrascosa F.M., Expósito J., Hedhili A., Olea N. (2015). Risk of female breast cancer and serum concentrations of organochlorine pesticides and polychlorinated biphenyls: a case–control study in Tunisia. Sci. Total Environ..

[195] Weisburger J.H. (2002). Comments on the history and importance of aromatic and heterocyclic amines in public health. Mutat. Res. Fund Mol. Mech. Mutagen.

[196] Lucchesi C.A., Vasilatis D.M., Mantrala S., Chandrasekar T., Mudryj M., Ghosh P.M. (2023). Pesticides and bladder cancer: mechanisms leading to anti-cancer drug chemoresistance and new chemosensitization strategies. Int. J. Mol. Sci..

[197] Koutros S., Lynch C.F., Ma X., Lee W.J., Hoppin J.A., Christensen C.H., Andreotti G., Freeman L.B., Rusiecki J.A., Hou L. (2009). Heterocyclic aromatic amine pesticide use and human cancer risk: results from the US Agricultural Health Study. Int. J. Cancer.

[198] Koutros S., Silverman D.T., Alavanja M.C., Andreotti G., Lerro C.C., Heltshe S., Lynch C.F., Sandler D.P., Blair A., Beane Freeman L.E. (2016). Occupational exposure to pesticides and bladder cancer risk. Int. J. Epidemiol..

[199] Amr S., Dawson R., Saleh D.a.A., Magder L.S., George D.M. St, El-Daly M., Squibb K., Mikhail N.N., Abdel-Hamid M., Khaled H. (2015). Pesticides, gene polymorphisms, and bladder cancer among Egyptian agricultural workers. Arch. Environ. Occup. Health.

[200] Kapeleka J.A., Sauli E., Ndakidemi P.A. (2021). Pesticide exposure and genotoxic effects as measured by DNA damage and human monitoring biomarkers. Int. J. Environ. Health Res..

[201] Ejaz S., Akram W., Lim C.W., Lee J.J., Hussain I. (2004). Endocrine disrupting pesticides: a leading cause of cancer among rural people in Pakistan. Exp. Oncol..

[202] Greenop K.R., Peters S., Bailey H.D., Fritschi L., Attia J., Scott R.J., Glass D.C., De Klerk N.H., Alvaro F., Armstrong B.K. (2013). Exposure to pesticides and the risk of childhood brain tumors. Cancer Causes Control.

[203] Van Maele-Fabry G., Gamet-Payrastre L., Lison D. (2017). Residential exposure to pesticides as risk factor for childhood and young adult brain tumors: a systematic review and meta-analysis. Environ. Int..

[204] Yousefi F., Asadikaram G., Karamouzian S., Abolhassani M., Pourghadamyari H., Moazed V., Khanjani N., Paydar P. (2022). Organochlorine and organophosphorus pesticides may induce brain cancer through oxidative stress. Toxicol. Ind. Health.

[205] Navas‐Acién A., Pollán M., Gustavsson P., Plato N. (2002). Occupation, exposure to chemicals and risk of gliomas and meningiomas in Sweden. Am. J. Ind. Med..

[206] Barsouk A., Thandra K.C., Saginala K., Rawla P., Barsouk A. (2021). Chemical risk factors of primary liver cancer: an update. Hepatic Med..

[207] Zhang H., Zhang R., Zeng X., Wang X., Wang D., Jia H., Xu W., Gao Y. (2022). Exposure to neonicotinoid insecticides and their characteristic metabolites: association with human liver cancer. Environ. Res..

[208] Haque A., Sahu V., Lombardo J.L., Xiao L., George B., Wolff R.A., Morris J.S., Rashid A., Kopchick J.J., Kaseb A.O. (2022). Disruption of growth hormone receptor signaling abrogates hepatocellular carcinoma development. J. Hepatocell. Carcinoma.

[209] Mnif W., Hassine A.I.H., Bouaziz A., Bartegi A., Thomas O., Roig B. (2011). Effect of endocrine disruptor pesticides: a review. Int. J. Environ. Res. Publ. Health.

[210] Yang Q., Salim L., Yan C., Gong Z. (2019). Rapid analysis of effects of environmental toxicants on tumorigenesis and inflammation using a transgenic zebrafish model for liver cancer. Mar. Biotechnol..

[211] Engel L.S., Zabor E.C., Satagopan J., Widell A., Rothman N., O'Brien T.R., Zhang M., Van Den Eeden S.K., Grimsrud T.K. (2019). Prediagnostic serum organochlorine insecticide concentrations and primary liver cancer: a case–control study nested within two prospective cohorts. Int. J. Cancer.

[212] Saad-Hussein A., Beshir S., Taha M.M., Shahy E.M., Shaheen W., Abdel-Shafy E.A., Thabet E. (2019). Early prediction of liver carcinogenicity due to occupational exposure to pesticides. Mutat. Res. Genet. Toxicol. Environ. Mutagen.

[213] Damalas C.A., Koutroubas S.D. (2016).

[214] Paumgartten F.J. (2020). Pesticides and public health in Brazil. Current Opinion in Toxicology.

[215] Greenlee A.R., Arbuckle T.E., Chyou P.-H. (2003). Risk factors for female infertility in an agricultural region. Epidemiology.

[216] Recio R., Robbins W.A., Borja-Aburto V., Moran-Martinez J., Froines J., Hernandez R., Cebrián M.E. (2001). Organophosphorous pesticide exposure increases the frequency of sperm sex null aneuploidy. Environ. Health Perspect..

[217] Chhillar S., Batra V., Kumaresan A., Kumar R., Pal A., Datta T.K. (2023). Acute exposure to organophosphorus pesticide metabolites compromises buffalo sperm function and impairs fertility. Sci. Rep..

[218] Oliva A., Giami A., Multigner L. (2002). Environmental agents and erectile dysfunction: a study in a consulting population. J. Androl..

[219] Hood R.B., Liang D., Chiu Y.-H., Sandoval-Insausti H., Chavarro J.E., Jones D., Hauser R., Gaskins A.J. (2022). Pesticide residue intake from fruits and vegetables and alterations in the serum metabolome of women undergoing infertility treatment. Environ. Int..

[220] Fortes C., Mastroeni S., Pilla M., Antonelli G., Lunghini L., Aprea C. (2013). The relation between dietary habits and urinary levels of 3-phenoxybenzoic acid, a pyrethroid metabolite. Food Chem. Toxicol..

[221] Chiu Y., Afeiche M., Gaskins A., Williams P., Petrozza J., Tanrikut C., Hauser R., Chavarro J. (2015). Fruit and vegetable intake and their pesticide residues in relation to semen quality among men from a fertility clinic. Hum. Reprod..

[222] Li J., Lin S., Wu J., Pei L., Shang X. (2023). Association between maternal exposure to chemical fertilizer and the risk of birth defects in a rural population in northern China: a population-based study. International Health.

[223] Yin S., Sun Y., Yu J., Su Z., Tong M., Zhang Y., Liu J., Wang L., Li Z., Ren A. (2021). Prenatal exposure to organochlorine pesticides is associated with increased risk for neural tube defects. Sci. Total Environ..

[224] Karlsson O., Svanholm S., Eriksson A., Chidiac J., Eriksson J., Jernerén F., Berg C. (2021). Pesticide-induced multigenerational effects on amphibian reproduction and metabolism. Sci. Total Environ..

[225] García J., Ventura M.I., Requena M., Hernández A.F., Parrón T., Alarcón R. (2017). Association of reproductive disorders and male congenital anomalies with environmental exposure to endocrine active pesticides. Reprod. Toxicol..

[226] Garry V.F., Harkins M.E., Erickson L.L., Long-Simpson L.K., Holland S.E., Burroughs B.L. (2002). Birth defects, season of conception, and sex of children born to pesticide applicators living in the Red River Valley of Minnesota, USA. Environ. Health Perspect..

[227] García A.M. (1998). Occupational exposure to pesticides and congenital malformations: a review of mechanisms, methods, and results. Am. J. Ind. Med..

[228] Cannarella R., Gül M., Rambhatla A., Agarwal A. (2023). Temporal decline of sperm concentration: role of endocrine disruptors. Endocrine.

[229] El-Baz M.A., Amin A.F., Mohany K.M. (2023). Exposure to pesticide components causes recurrent pregnancy loss by increasing placental oxidative stress and apoptosis: a case–control study. Sci. Rep..

[230] Crisostomo L., Molina V.V. (2002). Pregnancy outcomes among farming households of Nueva Ecija with conventional pesticide use versus integrated pest management. Int. J. Occup. Environ. Health.

[231] Arbuckle T.E., Savitz D.A., Mery L.S., Curtis K.M. (1999). Exposure to phenoxy herbicides and the risk of spontaneous abortion. Epidemiology.

[232] Mattila T., Santonen T., Andersen H.R., Katsonouri A., Szigeti T., Uhl M., Wąsowicz W., Lange R., Bocca B., Ruggieri F. (2021). Scoping Review—the association between asthma and environmental chemicals. Int. J. Environ. Res. Publ. Health.

[233] Islam J.Y., Hoppin J., Mora A.M., Soto-Martinez M.E., Gamboa L.C., Castañeda J.E.P., Reich B., Lindh C., De Joode B.V.W. (2023). Respiratory and allergic outcomes among 5-year-old children exposed to pesticides. Thorax.

[234] Balluz L.S., Philen R.M., Brock J., Falter K., Kiefer M., Hart R., Hill R.H. (2000). Health complaints related to pesticide stored at a public health clinic. Environ. Res..

[235] Omland O. (2002). Exposure and respiratory health in farming in temperate zones-a review of the literature. Ann. Agric. Environ. Med..

[236] Langley R.L. (2011). Consequences of respiratory exposures in the farm environment. N. C. Med. J..

[237] Faria N.M.X., Fassa A.G., Meucci R.D., Fiori N.S., Miranda V.I. (2014). Occupational exposure to pesticides, nicotine and minor psychiatric disorders among tobacco farmers in southern Brazil. Neurotoxicology.

[238] Amaral A.F. (2014).

[239] Azuma M., Nishioka Y., Aono Y., Inayama M., Makino H., Kishi J., Shono M., Kinoshita K., Uehara H., Ogushi F. (2007). Role of α1-acid glycoprotein in therapeutic antifibrotic effects of imatinib with macrolides in mice. Am. J. Respir. Crit. Care Med..

[240] Låg M., Øvrevik J., Refsnes M., Holme J.A. (2020). Potential role of polycyclic aromatic hydrocarbons in air pollution-induced non-malignant respiratory diseases. Respir. Res..

[241] Karimi P., Peters K.O., Bidad K., Strickland P.T. (2015). Polycyclic aromatic hydrocarbons and childhood asthma. Eur. J. Epidemiol..

[242] Delfino R.J. (2002). Epidemiologic evidence for asthma and exposure to air toxics: linkages between occupational, indoor, and community air pollution research. Environ. Health Perspect..

[243] Jenerowicz D., Silny W., Danczak-Pazdrowska A., Polanska A., Osmola-Mankowska A., Olek-Hrab K. (2012). Environmental factors and allergic diseases. Ann. Agric. Environ. Med..

[244] Klingbeil E., Hew K., Nygaard U.C., Nadeau K. (2014). Polycyclic aromatic hydrocarbons, tobacco smoke, and epigenetic remodeling in asthma. Immunol. Res..

[245] Wisnewski A.V., Redlich C.A. (2001). Recent developments in diisocyanate asthma. Curr. Opin. Allergy Clin. Immunol..

[246] Kim K.-H., Jahan S.A., Kabir E., Brown R.J. (2013). A review of airborne polycyclic aromatic hydrocarbons (PAHs) and their human health effects. Environ. Int..

[247] Chain E.P.o.C.i.t.F., Schrenk D., Bignami M., Bodin L., Chipman J.K., del Mazo J., Grasl‐Kraupp B., Hogstrand C., Hoogenboom L., Leblanc J.C. (2020). Risk to human health related to the presence of perfluoroalkyl substances in food. EFSA J..

[248] Smit L.A., Lenters V., Høyer B.B., Lindh C.H., Pedersen H.S., Liermontova I., Jönsson B.A., Piersma A.H., Bonde J.P., Toft G. (2015). Prenatal exposure to environmental chemical contaminants and asthma and eczema in school‐age children. Allergy.

[249] Baur X., Bakehe P. (2014). Allergens causing occupational asthma: an evidence-based evaluation of the literature. Int. Arch. Occup. Environ. Health.

[250] Collins J.J., Anteau S., Conner P.R., Cassidy L.D., Doney B., Wang M.L., Kurth L., Carson M., Molenaar D., Redlich C.A. (2017). Incidence of occupational asthma and exposure to toluene diisocyanate in the United States toluene diisocyanate production industry. J. Occup. Environ. Med..

[251] Koureas M., Tsakalof A., Tsatsakis A., Hadjichristodoulou C. (2012). Systematic review of biomonitoring studies to determine the association between exposure to organophosphorus and pyrethroid insecticides and human health outcomes. Toxicol. Lett..

[252] Rahman Z., Singh V.P. (2019). The relative impact of toxic heavy metals (THMs)(arsenic (As), cadmium (Cd), chromium (Cr)(VI), mercury (Hg), and lead (Pb)) on the total environment: an overview. Environ. Monit. Assess..

[253] Helaskoski E., Suojalehto H., Virtanen H., Airaksinen L., Kuuliala O., Aalto-Korte K., Pesonen M. (2014). Occupational asthma, rhinitis, and contact urticaria caused by oxidative hair dyes in hairdressers. Ann. Allergy Asthma Immunol..

[254] Rahman Z., Singh V.P. (2019). The relative impact of toxic heavy metals (THMs)(arsenic (As), cadmium (Cd), chromium (Cr)(VI), mercury (Hg), and lead (Pb)) on the total environment: an overview. Environ. Monit. Assess..

[255] North M.L., Takaro T.K., Diamond M.L., Ellis A.K. (2014). Effects of phthalates on the development and expression of allergic disease and asthma. Ann. Allergy Asthma Immunol..

[256] Koch H.M., Angerer J. (2011). Phthalates: biomarkers and human biomonitoring. Biomark. Hum. Biomonit.

[257] Ahmad M.F., Ahmad F.A., Zeyaullah M., Alsayegh A.A., Mahmood S.E., AlShahrani A.M., Khan M.S., Shama E., Hamouda A., Elbendary E.Y. (2023). Ganoderma lucidum: novel insight into hepatoprotective potential with mechanisms of action. Nutrients.

[258] Ren T.-Y., Fan J.-G. (2021). What are the clinical settings and outcomes of lean NAFLD?. Nat. Rev. Gastroenterol. Hepatol..

[259] Yang K.C., Hung H.-F., Lu C.-W., Chang H.-H., Lee L.-T., Huang K.-C. (2016). Association of non-alcoholic fatty liver disease with metabolic syndrome independently of central obesity and insulin resistance. Sci. Rep..

[260] Laothong U. (2016). Pesticides and non-alcoholic fatty liver disease. Thai Journal of Toxicology.

[261] Li M., Liu T., Yang T., Zhu J., Zhou Y., Wang M., Wang Q. (2022). Gut microbiota dysbiosis involves in host non-alcoholic fatty liver disease upon pyrethroid pesticide exposure. Environmental science and ecotechnology.

[262] Rives C., Fougerat A., Ellero-Simatos S., Loiseau N., Guillou H., Gamet-Payrastre L., Wahli W. (2020). Oxidative stress in NAFLD: role of nutrients and food contaminants. Biomolecules.

[263] Cano R., Pérez J.L., Dávila L.A., Ortega Á., Gómez Y., Valero-Cedeño N.J., Parra H., Manzano A., Véliz Castro T.I., Albornoz M.P.D. (2021). Role of endocrine-disrupting chemicals in the pathogenesis of non-alcoholic fatty liver disease: a comprehensive review. Int. J. Mol. Sci..

[264] Sule R.O., Condon L., Gomes A.V. (2022). A common feature of pesticides: oxidative stress—the role of oxidative stress in pesticide-induced toxicity. Oxid. Med. Cell. Longev..

[265] Lushchak V.I., Matviishyn T.M., Husak V.V., Storey J.M., Storey K.B. (2018). Pesticide toxicity: a mechanistic approach. EXCLI journal.

[266] Wahlang B., Hardesty J.E., Jin J., Falkner K.C., Cave M.C. (2019). Polychlorinated biphenyls and nonalcoholic fatty liver disease. Current opinion in toxicology.

[267] Clair H.B., Pinkston C.M., Rai S.N., Pavuk M., Dutton N.D., Brock G.N., Prough R.A., Falkner K.C., McClain C.J., Cave M.C. (2018). Liver disease in a residential cohort with elevated polychlorinated biphenyl exposures. Toxicol. Sci..

[268] Guvenius D.M., Hassanzadeh P., Bergman A., Norent K. (2002). Metabolites of polychlorinated biphenyls in human liver and adipose tissue. Environ. Toxicol. Chem.: Int. J..

[269] Tyagi S., Siddarth M., Mishra B.K., Banerjee B.D., Urfi A.J., Madhu S.V. (2021). High levels of organochlorine pesticides in drinking water as a risk factor for type 2 diabetes: a study in north India. Environ. Pollut..

[270] Miranda R.A., Silva B.S., de Moura E.G., Lisboa P.C. (2023). Pesticides as endocrine disruptors: programming for obesity and diabetes. Endocrine.

[271] Lin J.-Y., Yin R.-X. (2023). Exposure to endocrine-disrupting chemicals and type 2 diabetes mellitus in later life. Exposure and Health.

[272] Kumar V., Sharma N., Sharma P., Pasrija R., Kaur K., Umesh M., Thazeem B. (2023). Toxicity analysis of endocrine disrupting pesticides on non-target organisms: a critical analysis on toxicity mechanisms. Toxicol. Appl. Pharmacol..

[273] Sylvie Azandjeme C., Bouchard M., Fayomi B., Djrolo F., Houinato D., Delisle H. (2013). Growing burden of diabetes in sub-saharan Africa: contribution of pesticides?. Curr. Diabetes Rev..

[274] Tang M., Chen K., Yang F., Liu W. (2014). Exposure to organochlorine pollutants and type 2 diabetes: a systematic review and meta-analysis. PLoS One.

[275] Saldana T.M., Basso O., Hoppin J.A., Baird D.D., Knott C., Blair A., Alavanja M.C., Sandler D.P. (2007). Pesticide exposure and self-reported gestational diabetes mellitus in the Agricultural Health Study. Diabetes Care.

[276] Starling A.P., Umbach D.M., Kamel F., Long S., Sandler D.P., Hoppin J.A. (2014). Pesticide use and incident diabetes among wives of farmers in the Agricultural Health Study. Occup. Environ. Med..

[277] Hansen M.R., Jørs E., Lander F., Condarco G., Schlünssen V. (2014). Is cumulated pyrethroid exposure associated with prediabetes? A cross-sectional study. J. Agromed..

[278] Everett C.J., Thompson O.M. (2015). Association of DDT and heptachlor epoxide in human blood with diabetic nephropathy. Rev. Environ. Health.

[279] Everett C.J., Thompson O.M., Dismuke C.E. (2017). Exposure to DDT and diabetic nephropathy among Mexican Americans in the 1999–2004 national health and nutrition examination survey. Environ. Pollut..

[280] Juntarawijit C., Juntarawijit Y. (2018). Association between diabetes and pesticides: a case-control study among Thai farmers. Environ. Health Prev. Med..

[281] Sevim Ç., Çomaklı S., Taghizadehghalehjoughi A., Özkaraca M., Mesnage R., Kovatsi L., Burykina T.I., Kalogeraki A., Antoniou M.N., Tsatsakis A. (2019). An imazamox-based herbicide causes apoptotic changes in rat liver and pancreas. Toxicol Rep.

[282] Lind P.M., Lind L. (2018). Endocrine-disrupting chemicals and risk of diabetes: an evidence-based review. Diabetologia.

[283] Ruiz D., Becerra M., Jagai J.S., Ard K., Sargis R.M. (2018). Disparities in environmental exposures to endocrine-disrupting chemicals and diabetes risk in vulnerable populations. Diabetes Care.

[284] Rother H.-A., Hall R., London L. (2008). Pesticide use among emerging farmers in South Africa: contributing factors and stakeholder perspectives. Dev. South Afr..

[285] ElSayed N.A., Aleppo G., Aroda V.R., Bannuru R.R., Brown F.M., Bruemmer D., Collins B.S., Hilliard M.E., Isaacs D., Johnson E.L. (2023). 2. Classification and diagnosis of diabetes: standards of care in diabetes—2023. Diabetes Care.

[286] Owens K., Feldman J., Kepner J. (2010). Wide range of diseases linked to pesticides, database.

[287] Juntarawijit Y., Juntarawijit C. (2023). Pesticide exposure and rhinitis: a cross-sectional study among farmers in Pitsanulok, Thailand. F1000Research.

[288] Kimber I., Dearman R.J. (2010). An assessment of the ability of phthalates to influence immune and allergic responses. Toxicology.

[289] Odebeatu C.C., Taylor T., Fleming L.E., J Osborne N. (2019). Phthalates and asthma in children and adults: US nhanes 2007–2012. Environ. Sci. Pollut. Control Ser..

[290] Hoppin J.A., Jaramillo R., London S.J., Bertelsen R.J., Salo P.M., Sandler D.P., Zeldin D.C. (2013). Phthalate exposure and allergy in the US population: results from NHANES 2005–2006. Environ. Health Perspect..

[291] Jaakkola J.J., Knight T.L. (2008). The role of exposure to phthalates from polyvinyl chloride products in the development of asthma and allergies: a systematic review and meta-analysis. Environ. Health Perspect..

[292] Malvestio A., Bovenzi M., Hoteit M., Belloni Fortina A., Peserico A., Teresa Corradin M., Larese Filon F. (2011). p‐Phenylenediamine sensitization and occupation. Contact Dermatitis.

[293] Rafeeinia A., Asadikaram G., Moazed V., Darabi M.K. (2023). Organochlorine pesticides may induce leukemia by methylation of CDKN2B and MGMT promoters and histone modifications. Gene.

[294] A. Nguyen, Analysis of potential right-of-way environmental exposures and childhood leukemia: high voltage power lines, Plant Nurseries, and Pesticides, University of California, Los Angeles2023.

[295] Maryam Z., Sajad A., Maral N., Zahra L., Sima P., Zeinab A., Zahra M., Fariba E., Sezaneh H., Davood M. (2015). Relationship between exposure to pesticides and occurrence of acute leukemia in Iran. Asian Pac. J. Cancer Prev. APJCP.

[296] L. Mott, Our children at risk: the 5 worst environmental threats to their health, Natural Resources Defense Council1997.

[297] Weisenburger D.D. (2021). A review and update with perspective of evidence that the herbicide glyphosate (Roundup) is a Cause of non-Hodgkin lymphoma. Clin. Lymphoma, Myeloma & Leukemia.

[298] Acquavella J., Garabrant D., Marsh G., Sorahan T., Weed D.L. (2016). Glyphosate epidemiology expert panel review: a weight of evidence systematic review of the relationship between glyphosate exposure and non-Hodgkin’s lymphoma or multiple myeloma. Crit. Rev. Toxicol..

[299] Brusick D., Aardema M., Kier L., Kirkland D., Williams G. (2016). Genotoxicity Expert Panel review: weight of evidence evaluation of the genotoxicity of glyphosate, glyphosate-based formulations, and aminomethylphosphonic acid. Crit. Rev. Toxicol..

[300] Zhang L., Rana I., Shaffer R.M., Taioli E., Sheppard L. (2019). Exposure to glyphosate-based herbicides and risk for non-Hodgkin lymphoma: a meta-analysis and supporting evidence. Mutat. Res. Rev. Mutat. Res..

[301] Ewald J.A., Wheatley C.J., Aebischer N.J., Moreby S.J., Duffield S.J., Crick H.Q., Morecroft M.B. (2015). Influences of extreme weather, climate and pesticide use on invertebrates in cereal fields over 42 years. Global Change Biol..

[302] Hallmann C.A., Sorg M., Jongejans E., Siepel H., Hofland N., Schwan H., Stenmans W., Müller A., Sumser H., Hörren T. (2017). More than 75 percent decline over 27 years in total flying insect biomass in protected areas. PLoS One.

[303] Mehmood Y., Arshad M., Mahmood N., Kächele H., Kong R. (2021). Occupational hazards, health costs, and pesticide handling practices among vegetable growers in Pakistan. Environ. Res..

[304] Rajmohan K., Chandrasekaran R., Varjani S. (2020). A review on occurrence of pesticides in environment and current technologies for their remediation and management. Indian J. Microbiol..

[305] S. Arya, R. Kumar, O. Prakash, A. Rawat, A. Pant, Impact of insecticides on soil and environment and their management strategies, Agrochemicals in Soil and Environment: Impacts and Remediation, Springer2022, pp. 213-230.

[306] Dhuldhaj U.P., Singh R., Singh V.K. (2023). Pesticide contamination in agro-ecosystems: toxicity, impacts, and bio-based management strategies. Environ. Sci. Pollut. Control Ser..

[307] Loos R., Gawlik B.M., Locoro G., Rimaviciute E., Contini S., Bidoglio G. (2009). EU-wide survey of polar organic persistent pollutants in European river waters. Environ. Pollut..

[308] Brown C.D., Van Beinum W. (2009). Pesticide transport via sub-surface drains in Europe. Environ. Pollut..

[309] Helfrich L.A., Weigmann D.L., Hipkins P.A., Stinson E.R. (2009).

[310] Scholz N.L., Fleishman E., Brown L., Werner I., Johnson M.L., Brooks M.L., Mitchelmore C.L., Schlenk D. (2012). A perspective on modern pesticides, pelagic fish declines, and unknown ecological resilience in highly managed ecosystems. Bioscience.

[311] Hussain S., Siddique T., Saleem M., Arshad M., Khalid A. (2009). Impact of pesticides on soil microbial diversity, enzymes, and biochemical reactions. Adv. Agron..

[312] Rehana Z., Malik A., Ahmad M. (1995). Mutagenic activity of the Ganges water with special reference to the pesticide pollution in the river between Kachla to Kannauj (UP), India. Mutat. Res. Genet. Toxicol..

[313] Agarwal A., Prajapati R., Singh O.P., Raza S., Thakur L. (2015). Pesticide residue in water—a challenging task in India. Environ. Monit. Assess..

[314] Dwivedi S., Mishra S., Tripathi R.D. (2018). Ganga water pollution: a potential health threat to inhabitants of Ganga basin. Environ. Int..

[315] Ali S., Ullah M.I., Sajjad A., Shakeel Q., Hussain A. (2021). Environmental and health effects of pesticide residues, sustainable agriculture reviews 48: pesticide occurrence. Analysis and Remediation.

[316] Mitra A., Chatterjee C., Mandal F.B. (2011). Synthetic chemical pesticides and their effects on birds. Res J Environ Toxicol.

[317] Ahmad M.F., Ali M., Alsayegh A.R.A., Ahmad S., Alam N., Wahab S., Ali M.S., Athar M.T. (2021). A current novel perspective approach for coronavirus disease-2019 pandemic outbreak. \"J. Adv. Pharm. Technol. Research\"\" (JAPTR)\".

[318] Ahmad M.F., Wahab S., Ahmad F.A., Alam M.I., Ather H., Siddiqua A., Ashraf S.A., Shaphe M.A., Khan M.I., Beg R.A. (2021). A novel perspective approach to explore pros and cons of face mask in prevention the spread of SARS-CoV-2 and other pathogens. Saudi Pharmaceut. J..

[319] Aktar W., Sengupta D., Chowdhury A. (2009). Impact of pesticides use in agriculture: their benefits and hazards. Interdiscipl. Toxicol..

[320] Thieme T., Heimbach U., Müller A. (2010). Biocontrol-based Integrated Management of Oilseed Rape Pests.

[321] Şengül Demirak M.Ş., Canpolat E. (2022). Plant-based bioinsecticides for mosquito control: impact on insecticide resistance and disease transmission. Insects.

[322] Frangenberg A. (2000). Sustainable development of agricultural process and consequences of the implementation of Agenda 21. Integrated crop management as fundamental basis for sustainable production. Pflanzenschutz-Nachrichten Bayer.

[323] Baker B.P., Benbrook C.M., Iii E.G., Benbrook K.L. (2002). Pesticide residues in conventional, integrated pest management (IPM)-grown and organic foods: insights from three US data sets. Food Addit. Contam..

[324] Nwilene F.E., Nwanze K., Youdeowei A. (2008). Impact of integrated pest management on food and horticultural crops in Africa. Entomol. Exp. Appl..

[325] Chandler D., Davidson G., Grant W., Greaves J., Tatchell G. (2008). Microbial biopesticides for integrated crop management: an assessment of environmental and regulatory sustainability. Trends Food Sci. Technol..

[326] Kogan M. (1998). Integrated pest management: historical perspectives and contemporary developments. Annu. Rev. Entomol..

[327] Way M., Van Emden H. (2000). Integrated pest management in practice—pathways towards successful application. Crop Protect..

[328] Palacios Xutuc C. (2010).

[329] Van Boxstael S., Habib I., Jacxsens L., De Vocht M., Baert L., Van de Perre E., Rajkovic A., Lopez-Galvez F., Sampers I., Spanoghe P. (2013). Food safety issues in fresh produce: bacterial pathogens, viruses and pesticide residues indicated as major concerns by stakeholders in the fresh produce chain. Food Control.

[330] Heeb L., Jenner E., M.J. Cock (2019). Climate-smart pest management: building resilience of farms and landscapes to changing pest threats. J. Pest. Sci..

[331] Damalas C.A., Eleftherohorinos I.G. (2011). Pesticide exposure, safety issues, and risk assessment indicators. Int. J. Environ. Res. Publ. Health.

[332] Farrar J.J., Ellsworth P.C., Sisco R., Baur M.E., Crump A., Fournier A.J., Murray M.K., Jepson P.C., Tarutani C.M., Dorschner K.W. (2018). Assessing compatibility of a pesticide in an IPM program. Journal of Integrated Pest Management.

[333] Sharifzadeh M.S., Abdollahzadeh G., Damalas C.A., Rezaei R., Ahmadyousefi M. (2019). Determinants of pesticide safety behavior among Iranian rice farmers. Sci. Total Environ..

[334] Bhandari G., Atreya K., Yang X., Fan L., Geissen V. (2018). Factors affecting pesticide safety behaviour: the perceptions of Nepalese farmers and retailers. Sci. Total Environ..

[335] Rezaei R., Damalas C.A., Abdollahzadeh G. (2018).

[336] Saleem A., Haq I. (2003).

[337] Tariq M.I., Afzal S., Hussain I., Sultana N. (2007). Pesticides exposure in Pakistan: a review. Environ. Int..

[338] Bilal M., Iqbal H.M., Barceló D. (2019). Persistence of pesticides-based contaminants in the environment and their effective degradation using laccase-assisted biocatalytic systems. Sci. Total Environ..

[339] R. Neumann, Chemical Crop Protection Research and Development in Europe, Developments in Crop Science, Elsevier1997, pp. 49-55.

[340] Urech P. (1999). The agrochemical industry: its contribution to crop protection and environmental policy. Plant Pathol..

[341] Müller U. (2002). Chemical crop protection research. Methods and challenges. Pure Appl. Chem..

[342] Ye M., Beach J., Martin J.W., Senthilselvan A. (2013). Occupational pesticide exposures and respiratory health. Int. J. Environ. Res. Publ. Health.

[343] MacFarlane E., Carey R., Keegel T., El-Zaemay S., Fritschi L. (2013). Dermal exposure associated with occupational end use of pesticides and the role of protective measures. Safety and health at work.

[344] Fargnoli M., Lombardi M., Puri D., Casorri L., Masciarelli E., Mandić-Rajčević S., Colosio C. (2019). The safe use of pesticides: a risk assessment procedure for the enhancement of occupational health and safety (OHS) management. Int. J. Environ. Res. Publ. Health.

[345] Siriruttanapruk S., Anantagulnathi P. (2004). Occupational health and safety situation and research priority in Thailand. Ind. Health.

[346] Baker B.P., Green T.A., Loker A.J. (2020). Biological control and integrated pest management in organic and conventional systems. Biol. Control.

[347] Handford C.E., Elliott C.T., Campbell K. (2015). A review of the global pesticide legislation and the scale of challenge in reaching the global harmonization of food safety standards. Integrated Environ. Assess. Manag..

[348] Zhang W., Jiang F., Ou J. (2011). Global pesticide consumption and pollution: with China as a focus. Proceedings of the international academy of ecology and environmental sciences.

[349] Nordborg M., Davis J., Cederberg C., Woodhouse A. (2017). Freshwater ecotoxicity impacts from pesticide use in animal and vegetable foods produced in Sweden. Sci. Total Environ..

[350] S. Khoury, Pesticideland: Brazil's poison market, Revisiting Crimes of the Powerful, Routledge2018, pp. 174-187.

[351] Pelaez V., da Silva L.R., Araujo E.B. (2013). Regulation of pesticides: a comparative analysis. Sci. Publ. Pol..

[352] Cederberg C. (2013). Certification schemes (RTRS and ProTerra) in Brazilian soy: use of pesticides and cropping systems. SIK Institutet för livsmedel och bioteknik.

[353] S.A. Zayan, Impact of Climate Change on Plant Diseases and IPM Strategies, Plant Diseases-Current Threats and Management Trends, IntechOpen2019.

[354] Martin P., Ramalanjaona L., Truche C., Ballot R., Carozzi M., Pomeon T. (2023). Modelling the spatialisation of pesticide sales to monitor environmental policies in France. J. Clean. Prod..

